# NIR Spectroscopy for Non-Destructive Prediction of Greenhouse Gas Emissions and Global Warming Potential by Biomass Combustion

**DOI:** 10.3390/polym18091142

**Published:** 2026-05-06

**Authors:** Panmanas Sirisomboon, Prakash Gyawali, Jetsada Posom, Ravipat Lapcharoensuk, Bim Prasad Shrestha, Axel Funke

**Affiliations:** 1 Department of Biosystems and Agricultural Engineering, School of Engineering, King Mongkut’s Institute of Technology Ladkrabang, Bangkok 10520, Thailand; panmanas.si@kmitl.ac.th (P.S.); 66016181@kmitl.ac.th (P.G.); ravipat.la@kmitl.ac.th (R.L.); 2 Department of Agricultural Engineering, Faculty of Engineering, Khon Kaen University, Khon Kaen 40002, Thailand; 3 Nepal Technology Innovation Center (NTIC), Kathmandu University, Dhulikhel P.O. Box 6250, Nepal; 4 Department of Mechanical Engineering, School of Engineering, Kathmandu University, Dhulikhel P.O. Box 6250, Nepal; 5 Department of BioEngineering, University of Washington, William H. Foege Building 3720, 15th Ave NE, Seattle, WA 98195-5061, USA; 6 Institute of Catalysis Research and Technology (IKFT), Karlsruhe Institute of Technology (KIT), Hermann-von-Helmholtz-Platz 1, 76344 Eggenstein-Leopoldshafen, Germany; axel.funke@kit.edu

**Keywords:** NIR spectroscopy, greenhouse gas emission, global warming potential, higher heating value, sugarcane bagasse, biomass, combustion

## Abstract

Greenhouse gas (GHG) emissions from biomass combustion include carbon dioxide (CO_2_), methane (CH_4_) and nitrous oxide (N_2_O), which cause climate change and global warming. By measuring GHG emissions by biomass combustion, a potent protocol for the calculation of global warming potential (GWP), which is how much the global temperature has risen due to combustion processes, can be achieved, contributing to determining the mean reduction in global temperature rise and fostering a transition towards more sustainable energy systems. Additionally, warning can be given of the GHG and GWP risks associated with different species of biomass. This review includes the GHG emissions and GWP of biomass combustion and their measurement and estimation directly through biomass sample combustion, using unmanned aerial vehicles (UAVs) and satellite measurements of radiation interacting with atmospheric gases, or satellite-derived data and calculations according to IPCC guidelines. In addition, the relationship of lignocellulosic compounds and elements in biomass to HHV and GHG emissions is described. The key mechanism of molecular vibration of hydrogen bonds in biomass caused by NIR radiation related to GHG emissions is revealed and recorded regarding the possibility of using NIR spectroscopy for the prediction of GHG emissions and GWP. Calculation examples for sugarcane bagasse and other biomass species are shown. The comparative advantages and limitations of NIR spectroscopy with respect to other methods are included. These factors lead to elucidation of the possibility of using NIR spectroscopy for non-destructive prediction of GHG emissions. In this review, the feasibility of using NIR spectroscopy to evaluate GHG emissions, GWP and emission factors (EFs) as an alternative to IPCC estimation methods related to climate change by biomass combustion is confirmed. NIR spectroscopy is a novel methodology for predicting GHG emissions and GWP directly from intact chip or powder biomass spectral data without explicit gas measurement. This article records the essential spectroscopic knowledge of biomass polymer valorization that is of value in polymer science.

## 1. Greenhouse Gas (GHG) Emissions and Global Warming Potential (GWP)

### 1.1. GHG Emissions and GWP of Biomass Combustion

The need to coordinate global policies with the Sustainable Development Goals (SDGs) to achieve sustainable economic growth with the fewest possible environmental risks [1] is important for every spatial dimension of our world. Arif et al. stated on the basis of a study of the relationship between CO_2_ emissions caused by transportation and the inflow of foreign investment for developing economies (D-8 countries) that developing countries need to take a comprehensive and varied approach to achieve green economic growth by utilizing renewable energy to lessen environmental effects and make the environment more environmentally friendly [1].

The current fossil fuel crisis, increasing cost of fuel and rising environmental air pollution concerns have fostered the development of biomass resources as an alternative energy source. Energy plays a crucial role in the global economy and has a significant impact on a country’s economic standing. Through Advanced Energy Technologies [2], Thailand has its own reserves of fossil fuels in the form of coal, natural gas and crude oil, but they are significantly lower than those of the world leaders. The share of coal is 0.1% of the world’s total, that of natural gas is 0.08%, and that of oil is 0.02% [3]. In terms of tons of oil equivalent, according to 2024 data, the conventional proved reserves by fuel type were 82.4% for coal, 13.8% for natural gas, and 3.8% for oil. Bioenergy plays a significant role in Thailand’s power generation, contributing about 70% to all renewable electricity produced in 2020, followed by solar photovoltaics (solar PV) (11.4%), hydropower (10.4%), and wind energy (8.1%). However, the country does not completely use its overall renewable energy potential. In its Alternative Energy Development Plan (AEDP) 2018–2037, Thailand has set a new renewable energy target of 30% of total final energy consumption and meeting about 34% of net national electricity demand from renewable sources by 2037. In addition, the plan aims to achieve the following renewable energy capacities by 2037: 15.6 GW from solar, 5.8 GW from biomass, 3 GW from wind, 3 GW from hydropower, and 0.9 GW from waste [4]. Regarding energy-related carbon dioxide (CO_2_) emissions, in 2023, Thailand generated 244 Mt CO_2_, which was 0.7% of global emissions and represented a 59% change since 2000 [5].

Biomass combustion, as an energy source, offers several benefits in terms of greenhouse gas (GHG) emissions and global warming potential (GWP). Firstly, biomass is considered a carbon-neutral energy source. During its growth, biomass absorbs CO_2_ from the atmosphere through photosynthesis [6]. When it is combusted for energy, the CO_2_ released is roughly equivalent to the amount absorbed during its growth, resulting in a closed carbon cycle [6]. This significantly contrasts with fossil fuels, which release carbon stored for millions of years, adding new CO_2_ to the atmosphere. Utilizing biomass for energy can help mitigate climate change by reducing net GHG emissions when sustainably managed.

Moreover, biomass combustion can have a lower GWP compared to traditional fossil fuels. The GWP of a substance measures how much heat a GHG traps in the atmosphere over a specific period, typically 100 years, compared to the heat trapped by CO_2_. Biomass combustion often results in lower emissions of methane (CH_4_) and nitrous oxide (N_2_O), both potent GHGs. Additionally, modern biomass energy technologies have advanced to enhance combustion efficiency (Cr) and reduce particulate emissions. By replacing fossil fuels with biomass, we can reduce the overall GWP associated with energy production, contributing to a reduction in global temperature rise and fostering a transition towards more sustainable energy systems.

According to the U.S. Environmental Protection Agency (EPA) report on “Emissions from Wood-Fired Combustion Equipment”, wood biomass combustion emits about 1.8 kg of CO_2_ per kg of dry wood burned, with additional emissions of 0.015 kg of CH_4_ and less than 0.001 kg of CO_2_ per GJ of energy produced [7]. Using the Intergovernmental Panel on Climate Change (IPCC) Fifth Assessment Report (AR5) GWP values, the total GWP for wood biomass is approximately 2.518 kg CO_2_-equivalent per GJ of energy [8]. The total GWP for non-wood biomass, using higher values for a conservative estimate, is approximately 5.39 kg CO_2_-equivalent per GJ. These figures highlight the lower GWP of wood biomass compared to non-wood biomass, underscoring the importance of efficient combustion technologies and sustainable management practices to minimize environmental impacts [9]. 

The International Energy Agency (IEA) [10] reported in 2024 that the world total energy supply is 654 EJ, of which modern solid bioenergy contributes 37 EJ (about 5.7%). The IEA estimated that by 2035 the total energy supply will have increased to 744 EJ and that the modern solid bioenergy supply will have risen to 47 EJ (around 6.3%), representing an absolute increase of 10 EJ and a compound annual growth rate (CAGR) of about 2.2% per year [10]. By 2040, modern solid bioenergy will have reached 51 EJ (6.6% of total 777 EJ). By 2050, the total energy supply will have grown to 838 EJ, while modern solid bioenergy will have reached 59 EJ (approximately 7.0%), an increase of 22 EJ from 2024 and a CAGR close to 1.9% per year [10]. Regarding the world’s total final energy consumption, in 2024, it was 453 EJ, of which solid bioenergy accounted for 35 EJ, representing about 7.7% [10]. Solid bioenergy is estimated to increase only slightly over time, reaching 36 EJ in 2035, 37 EJ in 2040, and 38 EJ in 2050, and overall final consumption is predicted to grow faster, from 453 EJ in 2024 to 599 EJ in 2050 (32.2%), while the share of solid bioenergy is predicted to gradually decrease to around 6.3% by 2050 [10]. The long-term growth rate of solid bioenergy is very low, with a CAGR of about 0.3% per year between 2024 and 2050 [10]. The IEA Net Zero Emissions by 2050 Scenario (NZE Scenario) translates the 1.5 °C goal into a global pathway for the energy sector [10]. Global energy-related CO_2_ emissions amounted to 38 gigatonnes (Gt) in 2024, and according to the NZE Scenario, emissions will have fallen by nearly 55% by 2035 (~18 Gt) [10]. Yet the increase in long-term global average temperature is predicted to exceed 1.5 °C by around 2030 and to peak at 1.65 °C by around 2050. The NZE Scenario achieves the COP28 goals of doubling efficiency and tripling renewables capacity by 2030, and it meets the Paris agreement goal of holding warming well below 2 °C throughout the 21st century. Emission levels depend on the type of fuel, fuel consumption, and the efficiency of combustion equipment. The issue of harmful emissions from the combustion of raw and torrefied biomass primarily concerns wood and focuses on the measurement of CO, NO, CO_2_, CH_4_, and particulate matter [11,12,13,14]. The largest source of GHG emissions other than the energy sector is agriculture, forestry and other land use (AFOLU), which produced between 10 and 12 Gt CO_2_-eq net GHG emissions in recent years. CO_2_ emissions from AFOLU were around 5–6 Gt CO_2_, and N_2_O and CH4 emissions were around 5–6 Gt CO_2_-eq [15,16]. AFOLU emissions are emissions from anthropogenic activities and do not include CO_2_ emissions removal from the atmosphere by natural land sinks [14]. Energy sector GHG emissions include CO_2_ emissions from fuel combustion plus fugitive and vented methane, and nitrous oxide (N_2_O) emissions from the energy and industry sectors [16]. Cumulative global energy-related and industrial process CO_2_ emissions between 2020 and 2050 amount to just over 460 Gt in the NZE. Assuming parallel action to address CO_2_ emissions from agriculture, forestry and other land use (AFOLU) over the period to 2050 would result in around 40 Gt CO_2_ from AFOLU. This means that total CO_2_ emissions from all sources—some 500 Gt CO_2_—are in line with the CO_2_ budgets included in the IPCC SR1.5, which indicates that the total CO_2_ budget from 2020 consistent with providing a 50% chance of limiting warming to 1.5 °C is 500 Gt CO_2_ [16,17].

Furthermore, the risks associated with the combustion of different biomass species must be carefully managed to mitigate GHG emissions and GWP. For wood biomass, sustainable forestry practices and advancements in combustion technology are essential to maximize carbon neutrality and reduce emissions of CH_4_ and N_2_O. For non-wood biomass, selecting species with favorable combustion characteristics, employing pretreatment methods like drying and pelletizing, and using efficient combustion systems can help reduce emissions and improve overall sustainability. Addressing these risks through targeted strategies will enhance the climate benefits of both wood and non-wood biomass energy, supporting the transition to more sustainable and low-GWP energy systems.

Though biomass is a carbon-neutral fuel, the combustion of biomass constitutes a notable source of GHG emissions, playing a role in the phenomenon of climate change [18,19]. Accurately quantifying these emissions is crucial for developing effectiveness, mitigation strategies and understanding the environmental impact of biomass utilization. Traditional methods for determining GHG emissions and GWP from biomass combustion involve complex and time-consuming processes, often requiring specialized equipment and expertise [20]. Attracting the interest of diverse researchers and decision-makers, this matter has led to climate instability, deterioration in air quality, public health issues, and changes in ecosystems. The IPCC has evaluated the consequences of global warming at a 1.5 °C scale and delineated pathways pertinent to GHG emissions [17]. GHG emissions and GWP can be estimated by procedures of the IPCC using HHV in biomass. These procedures require sophisticated protocols, time-consuming calibration, sample preparation, experts and chemicals, with a high cost per sample and long timeframes, such that they are not economical or environmentally friendly. Rapid, reliable and non-destructive instruments are required to substitute the traditional methods.

However, the carbon neutrality of biomass combustion is highly conditional, as land-use change (LUC), temporal carbon debt, supply chain emissions, and combustion inefficiencies can substantially offset or even reverse the assumed climate benefits. These issues, LUC [21,22,23], carbon debt and payback time [24,25,26], feedstock supply chain emissions [27,28,29], and combustion inefficiencies (CH_4_ and N_2_O formation), were investigated and discussed thoroughly at the beginning of the 20th century (2001–2012) [30,31,32]. Biogenic CO_2_ is treated as zero in the energy sector, but this depends on carbon stock change [33,34,35].

In addition to direct combustion emissions, biomass systems incur GHG emissions throughout the supply chain, including harvesting, processing, and transportation. Life cycle assessments (LCAs) incorporating satellite-derived carbon flux data indicate that these upstream emissions can significantly reduce or negate the climate benefits of bioenergy systems [36,37]. Depending on feedstock type and ecosystem characteristics, carbon payback periods may range from a few years to several decades, during which biomass systems can act as a net source of GHGs [38]. LUC represents one of the most significant sources of GHG emissions associated with bioenergy systems. Conversion of forests or natural ecosystems for bioenergy feedstock production leads to substantial losses in aboveground biomass and soil organic carbon. Recent satellite-based analyses have quantified these losses at regional and global scales, demonstrating that LUC emissions can exceed the carbon savings from biomass substitution for fossil fuels [39,40]. The concept of carbon debt highlights the temporal imbalance between immediate emissions from biomass combustion and delayed carbon sequestration through regrowth. Combustion inefficiencies further compromise the carbon neutrality of biomass by increasing emissions of non-CO_2_ GHGs, such as CH_4_ and N_2_O; given high GWP, even small increases in these emissions can substantially elevate the overall climate impact of biomass combustion. Recent satellite-based fire emission studies confirm that combustion conditions strongly influence emission factors and resulting GHG outputs [41]. Table 1 shows the related factors affecting carbon neutrality of biomass combustion investigated by some researchers.

### 1.2. Direct Measurement and Estimation of GHG Emissions in Biomass Sample Combustion and Its GWP

Direct Measurement of Emissions
Laboratory Biomass Burning Experiment and Instrument Set-up

Gilman et al. [42] burned biomass inside large burn chambers (12.5 × 12.5 × 20 m hight), whose main components included (1) a fuel bed under an emissions-entraining hood, (2) an exhaust stack, and (3) an elevated sampling platform surrounding the exhaust stack 17 m above the fuel bed [42,43]. Each fuel sample was arranged on the fuel bed in a manner that mimicked their natural orientation and fuel loading when possible and was ignited using a small propane torch [42,43]. During each fire, the burn chamber was slightly pressurized with outside air conditioned to a temperature and relative humidity similar to those of the ambient air inside the burn chamber [42]. The subsequent emissions were entrained by the pre-conditioned ambient air and continuously vented through the top of the exhaust stack [42]. The residence time of emissions in the exhaust stack ranged from ∼5 to 17 s depending on the flow and/or vent rate [42]. Each burn lasted approximately 20–40 min from ignition to natural extinction [42].

In Gilman et al. [42], a comprehensive suite of instruments was used to quantify the emissions of over 200 organic gases, including CH_4_ and nine inorganic gases, including CO, CO_2_, and NO_x_, from 56 laboratory burns of 18 different biomass fuel types common in the southeastern, southwestern, or northern USA. A gas chromatograph–mass spectrometry (GC-MS) instrument provided extensive chemical detail of discrete air samples collected during a laboratory burn and was complemented by real-time measurements of organic and inorganic species via an open-path Fourier transform infrared spectroscopy (OP-FTIR) instrument and three different chemical ionization mass spectrometers. These measurements were conducted at the US Department of Agriculture’s Fire Sciences Laboratory in Missoula, MT, USA.

The GC-MS instrument and the proton-transfer-reaction mass spectrometry (PTR-MS) instrument were located in a laboratory adjacent to the burn chamber. The proton-transfer-reaction ion-trap mass spectrometry (PIT-MS) instrument, negative-ion proton-transfer chemical ionization mass spectrometry (NI-PT-CIMS) instrument, and OP-FTIR optical spectroscopy instrument were located on the elevated platform inside the burn chamber. The details of the instruments, sample gas inlets and sample lines, including dimensions and locations, and sampling inlet residence times and instrument response times are given in Gillman et al. [42].

Laboratory-Scale Boiler Using Biomass Burning Experiment and Instrument Set-up

**Figure 1:**
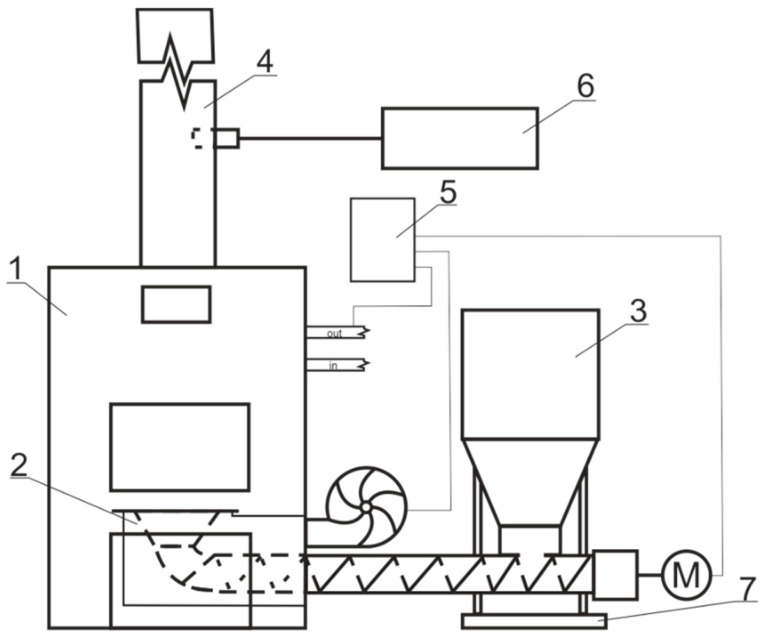
Scheme of boiler stand: 1—test boiler, 2—furnace, 3—pellet reservoir, 4—chimney, 5—boiler controller, 6—exhaust gas analyzer, 7—scales [44].

Wasilewski et al. [44] tested the combustion of selected types of agricultural biomass pellets (rape straw pellets and wood pellets) using a laboratory-scale boiler for measuring greenhouse gas emission levels during combustion, where the components of the test stand were (1) a 25 kW boiler which was equipped with (2) a furnace, to which fuel was fed from (3) a reservoir in an automated way. The furnace had (4) a chimney for flue gas to pass out to the environment. Boiler operation was controlled by (5) a programmed electronic controller. A diagram of the boiler stand is shown in Figure 1. The amount of fuel fed for combustion as well as the amount of air required for proper combustion was automatically selected by the controller, based on the results of measurements of the oxygen content in the flue gas provided by the lambda probe and the temperature sensor at the boiler outlet. The combustion test of the pellets lasted for 1 h. The fuel consumption was determined by weighing the fuel fed into the reservoir before and after the test for each fuel. The fuel mass flux for wood pellets was 6.15 kg h^−1^ and for rapeseed straw pellets it was 7.63 kg h^−1^. The flue gas temperature was 138 °C and 134 °C, respectively. The concentrations of nitrogen oxides (NO_x_), CO_2_, and CH_4_ were measured using the Testo 350 [44]. The Testo 350 is a portable flue gas analysis system for the measurement of exhaust gas emissions.

Laboratory-Scale Furnace for Biomass Pellet Burning Experiment and Instrument Set-up

The prototype pellet furnace with a 7–32 kW capacity used in Roy et al. [45] consists of (1) a hopper/bin with a capacity for 140 kg of pellets and a motor-controlled feed auger to introduce the pellets into the burn pot, (2) a control panel to control the feed rate (2–6 kg h^−1^ was the range tested), (3) an ash receiver to gather the ash produced in the combustion with an automatic ash-removal auger, (4) a burn pot with a rotating agitator, (5) a hot-water heat exchanger to collect the heat of combustion (the input thermal energy at the feed rates ranged from 10 to 31 kW), (6) an induced draft fan to control draft, and (7) a double-walled stainless steel chimney connected to the flue pipe from the furnace outlet.

At each fuel feed rate, the performance was tested under steady-state conditions, considered when there was almost no change in flue gas temperature or emission parameters. It took almost an hour to reach the steady-state condition. Upon stable flue temperatures, gas emissions were measured using a constant-flow flue gas analyzer for O_2_, CO, NO, NO_2_ and SO_2_ and a constant temperature (Unigas 3000+, Eurotron Instruments Srl, Sesto S. Giovanni, Milan, Italy). The sampling probe was placed about 8 cm before the induced draft fan. A water trap and a line filter separate water particles and particulates respectively from flue gases before sending these to the detection chamber. The analyzer measures the concentrations of different gases on a dry basis. The measurement principles of the gas analyzers are electrochemical for O_2_, CO, NO, NO_2_ and SO_2_, and calculated for CO_2_ and NO_x_. The NO_x_ concentration is calculated by the analyzer as the simple sum of measured NO and NO_2_ concentrations. Emissions data on CO, SO_2_, NO, NO_2_ and NO_x_ are corrected to 10% reference O_2_ in the flue gases.

A multiple thermocouple calibrator (ALTEK Model 322-1, Altek Industries Corp, Rochester, New York, USA) with four switchable thermocouple types (J, K, T and E) was used to measure the bed and maximum furnace temperatures, and the K-type was used. The ash was analyzed for its agglomeration in each test condition using variable pressure scanning electron microscope (VP-SEM) (Hitachi S-3000N, Hitachi High-Tech Corporation, Minato-ku, Tokyo, Japan) with a magnification range of 5–300,000. In every test case, different data were obtained for three repetitions for the calculation of the average value ± standard error.

Estimation of Emissions
Field Biomass Burning Experiment and Instrument Set-up

In most developing countries and underdeveloped countries, annual field clearing by biomass burning is generally carried out at the end of the dry season, and the clearing includes traditional slash-and-burn methods to clear plant biomass (shrubs, plant regeneration and crop residues) [46].

Bougma et al. [46] established experimental sites required for a survey to observe farmers’ land management practices, which led to the identification of representative sites relevant to biomass assessment and real-time burning experiments. The cropland classification and categories were selected based on IPCC criteria and their spatial distribution and accessibility, for instance, land converted to cropland (for example, less than 20 years old), cropland that has remained as cropland (for example, more than 20 years old), and so on. The experimental plots were established in each cropland category, and data were collected in two steps: biomass assessment in croplands and related sampling determinations for pre- and post-fire biomasses. A dimension of plot should not be less than 100 m × 100 m (10,000 m^2^), with 10 to 15 plots per cropland category, for the assessment of biomass [46]. The variability of different biomasses in terms of crop residues, plant regeneration and foliage from defoliation of agroforestry trees (usually located under the tree canopy), was measured within each plot: subplots of 25 m^2^ (5 m × 5 m) at least 10 m apart were set up, with three subplots located under tree canopies and three located outside. At the site level, a minimum distance of 1 km was observed among the main plots to account for the variation in soil types, crop varieties and plant species. The information used for the determination of tree density and tree canopy cover per plot total, including the number of adult trees and their morphological traits such as total height, stem diameter at breast height, and small and large crowns, were measured.

During the clearing season, Bougma et al. [46], for existing fuel biomass in fields, recommended harvesting and sun-drying it in situ for at least 1–2 weeks before burning to ensure that most of the biomass is potentially burnable. The dry fuel biomass assessed in the study included dead wood, crop residues, dead leaves and grass. Diameters of fuel components were randomly measured using vernier calipers. The total dry biomass (pre-fire biomass) in each subplot was separately weighed using an electronic balance (weight = 0–5 kg, with precision = 1 g) according to fuel size, i.e., fine fuel and large fuel. Large pieces of dead wood (diameter > 2.5 cm) were removed from the experiment as they are generally harvested and used as domestic firewood. After the fuel biomass was weighed, experimental fires were set on the investigated fields. The experiment had to account for farmers’ practices, where all biomass components from the subplots (under and outside the canopy) were mixed, and two composite biomass samples of 2 kg were taken to be burned separately on a steel tray of 4 m^2^ (2 m × 2 m) for determination of the carbon content. Fires were ignited in the direction of the wind, as practiced by farmers, between 4 and 5 pm using a bunch of dried grass biomass to ensure rapid linear ignition. Once they had sufficiently cooled, after each experimental fire, the residues left, i.e., all biomass post-fire (ash, charcoal and unburned fuels) remains, on the steel tray were collected and weighed to determine the amount of biomass consumed.

In the case of Bougma et al. [46], 96 experimental fires were conducted: 2 climatic zones × 2 sites × 2 cropland categories × 6 plots × 2 fire experiments, resulting in 96 composite samples pre-fire and post-fire. Pre-fire composite samples consisted of 0.1 kg of biomass randomly collected from 36 plots, while post-fire composite samples consisted of 0.05 kg of biomass randomly collected from 36 experiments. Possible unburned and charcoal fuels were separated according to the type of fuel components, as mentioned above, to estimate the fuel loss of each component. All composite samples collected were brought to the laboratory for moisture content and carbon content determination.

The details of the data analysis for carbon content (%), fuel biomass (t ha^−1^), moisture content (%), carbon loss (%), combustion completeness (%), carbon remaining post-fire (%), carbon emissions (t ha^−1^) and gas emission factors (g kg^−1^) can be explored in Bougma et al. [46].

### 1.3. IPCC Guidelines for Using Higher Heating Value of Biomass for GHG Emissions and GWP Calculation

The main GHG emissions from biomass combustion include CO_2_, CH_4_ and N_2_O, which effect climate change and cause global warming. The ability to evaluate the GHG emissions of different species of biomass can be extended beyond the immediate scientific realm, manifesting as a catalyst for informed decision-making and climate change mitigation. The IPCC was established in 1988, and protocols for the calculation of GHG emissions and GWP have been developed.

The calculation method provided by the IPCC for the GHG emissions of CO_2_, CH_4_, and N_2_O in biomass combustion is presented in Equation (1):

GHG (e.g., CO_2_, or CH_4_, or N_2_O) Emissions (kg) =Mass of biomass sample (kg) × HHV of biomass sample (TJ kg^−1^) × EF of corresponding GHG (kg TJ^−1^) (1)

GHG emissions (kg) represent the total amount of emissions produced. The mass of biomass samples (kg) refers to the total mass of the biomass sample burnt. The high heating value (HHV) (TJ kg^−1^) is the total amount of energy in TJ obtained from 1 kg of biomass completely burnt in a bomb calorimeter without deduction of the energy obtained in the form of the heat of vapor (biomass vapor evaporated during biomass burning) condensation [47]. According to the IPCC 2021 Guidelines, default GHG EFs (in kg TJ^−1^) for wood and woody biomass combustion are 112 kg CO_2_ TJ^−1^ (non-biogenic CO_2_ only), 30 kg CH_4_ TJ^−1^, and 4 kg N_2_OTJ^−1^, expressed based on the energy content of the fuel burned [48].

Using the GWP values recommended by the IPCC (2021) Sixth Assessment Reports (AR6) [48], 1 for CO_2_, 29.8 for CH_4_, and 273 for N_2_O, the total GWP was computed for 100 years using the following equation:

GWP total (kg CO_2_eq) = (1 × CO_2_ Emission) + (29.8 × CH_4_ Emission) + (273 × N_2_O Emission) for 100 years (2)

A GHG EF (kg TJ^−1^) for a specific biomass, the ratio of the mass of gas emitted to the unit of fuel burned energy, can be evaluated using Equation (3), GHGs including CO_2_, CH_4_, and N_2_O [49]:

EFx = Ex/E(3)

where EFx is the emission factor of gas x (kg TJ^−1^), Ex is the mass of gas x emitted, and E is the energy content of the fuel burned (TJ), where HHV is used for as-received biomass and LHV is used for dried biomass.

In another formula, Equation (4) can be used to calculate the EF of gross CO_2_ emissions for complete biomass combustion if the carbon content (CC) of the biomass is known [50].

EF of CO_2_ gross = C_f_ × CC × OF × (44/12) (4)

where EF of CO_2_ gross is the CO_2_ gross EF (kg TJ^−1^), C_f_ is the combustion efficiency (default = 1 for complete combustion), CC is the carbon content (kg TJ^−1^), OF is the fraction of carbon oxidized (usually = 1) and 44/12 is the molecular weight ratio of CO_2_ (44) to C (12), and CC can be calculated using Equation (5) [50].

CC = Carbon content (decimal) in biomass × mass of biomass (kg)/HHV (TJ kg^−1^ of biomass) (5)

The EF of CO_2_ calculated using Equations (3)–(5) represents gross CO_2_ emissions. In the IPCC Guidelines, CO_2_ from biomass combustion is reported as zero in the energy sector [49] because it is already accounted for in the agriculture, forestry and other land use (AFOLU) sector under land-use change or removals, where all carbon in wood (biomass), which is biogenic carbon—carbon absorbed from the atmosphere by a tree during growth [49]. This is the principle of the IPCC assumption of a carbon-neutral cycle: CO_2_ emitted during combustion is considered offset by CO_2_ absorbed during biomass growth [49,50].

The EF of biomass in the unit of kg CO_2_eq per metric ton of biomass burned can be calculated using Equation (6):

EF of biomass (kg CO_2_eq [metric ton of fuel burned]^−1^) = Sum [GWP of GHG (kg CO_2_eq) × GHG Emission (kg [ton of fuel burned]^−1^)] = [(1 × CO_2_ Emission) + (29.8 × CH_4_ Emission) + (273 × N_2_O Emission)] × 1000 kg (metric ton) of fuel burned = GWP total of 1 kg biomass (kg CO_2_eq) × 1000 kg (metric ton) of fuel burned (6)

### 1.4. GHG Emission Estimation from Biomass Combustion of Power Plant Using Unmanned Aerial Vehicles (UAVs)

The following systems are used specifically for the measurement of anthropogenic emissions of CO_2_ from fossil fuel combustion, where atmospheric measurements of CO_2_ mole fractions are crucial. However, they can be applied for biomass combustion in factories, e.g., electricity plants, using biomass as fuel when UAV data are calibrated with the gross bionic CO_2_, as the carbon-neutrality offset of biomass combustion is highly conditional due to land-use change (LUC), temporal carbon debt, supply chain emissions, and combustion inefficiencies.

Novel top-down carbon monitoring methods, including space-based, aerial and ground-based observations, are tantalizing alternatives to more reliable data sources of carbon emissions as well as sinks [51]. UAV-based carbon monitoring is an upcoming field due to its lower costs, higher spatial–temporal resolution and operability in a wider range of weather conditions compared to satellite observations [51].

Yang et al. [52] introduced a newly developed measurement-fed-perception self-adaption Low-cost UAV Coordinated Carbon Observation Network (LUCCN) prototype. The LUCCN primarily consists of two categories of instruments for ground-based and UAV-based in situ measurement. The GMP343, a low-cost non-dispersive infrared (NDIR) carbon dioxide (CO_2_) sensor, was used in both ground-based and UAV-based instruments. The first integrated measurement campaign took place in Shenzhen, China, 4 May 2023. During the campaign, LUCCN’s UAV component presented significant data-collecting advantages over its ground-based counterpart owing to the relatively high altitudes of the point emission sources, which were especially obvious at a gas power plant in Shenzhen. The emission flux was calculated by a cross-sectional flux (CSF) method, the results of which differed from the Open-Data Inventory for Anthropogenic Carbon dioxide (ODIAC). The CSF result was slightly larger than the others owing to the low sampling rate of the whole emission cross section.

The LUCCN system was tested using UAVs equipped with NDIR CO_2_ sensors, which can make measurements in the air with a faster response and can intelligently navigate themselves within areas of greater interest; with the help of lighter NDIR payloads and multiple UAVs flying in synchrony, larger cross sections or areas can be covered, maximizing the acquisition of information on emission plumes [51]. Zhao et al. [51] first introduced a design for a miniaturized NDIR payload which is compatible with a lightweight UAV to achieve a single system weighing less than 4 kg and then compared it against another NDIR payload to determine its accuracy (−0.82 ppm and −0.44 ppm) and precision (0.12 ppm and −0.03 ppm) in measurements of two vertical concentration profiles. Finally, four UAVs were deployed on two major anthropogenic emission source monitoring campaigns with coordinated flight patterns to fully understand the benefit of multi-UAV carbon monitoring; the two flight patterns and computed power plant emission rates were determined via the cross-sectional flux method. Every flight successfully captured the concentration enhancements downwind of the power plants; the single-sectional flight pattern measured an emission rate of 10ktCO_2_ day^−1^, which is 44% less than that reported monthly; and the multi-sectional flight pattern yielded relatively varied results (8.5 ktCO_2_ day^−1^ on average, which is 54% less) for the respective sections due to wind underestimations, plume section undersampling and wind fluctuations.

Ren et al. [53] present a multivariable linear regression (MLR) calibration method for NDIR CO_2_ sensors in a low-cost carbon monitoring network, with data collected in a temperature- and pressure-controlled laboratory, and evaluated the calibration method with long-term observational data collected at the Xinglong Atmospheric Background Observatory. The data collected were compared with high-accuracy cavity ring-down spectrometer (Picarro) data, and the results show that the MLR calibration incorporating temperature, pressure, and relative humidity can reduce the mean absolute bias from 5.218 ppm to 0.003 ppm, with root mean square errors (RMSEs) within 2.1 ppm after calibration [53]. The field observations provided the RMSE, which was reduced from 8.315 ppm to 2.154 ppm, and the bias decreased from 39.170 ppm to 0.018 ppm [53]. The calibrated data can effectively capture the diurnal variation in CO_2_ mole fractions, and about 10 days of co-located reference data are sufficient to obtain reliable measurements [53].

The protocol for obtaining GHG emissions using UAV data collection is described in detail in Yang et al. [51], Zhoa et al. [52] and Ren et al. [53].

### 1.5. GHG Emission Estimation from Biomass Burning Using Satellite Measured Radiation Interacting with Atmospheric Gases or Satellite-Derived Data

Advances in satellite observations and atmospheric inversion modeling have enabled more accurate, spatially resolved estimates of GHG emissions from biomass burning at a large scale on earth. These convey the fact of substantial variability in emissions across regions and time, challenging the assumption of uniform carbon neutrality.

Kuze et al. [54] shared a brief overview of the first greenhouse gases observing satellite (GOSAT), its scientific requirements, instrument designs, hardware performance, on-orbit operation, and data processing. The Japanese GOSAT monitors CO_2_ and CH_4_ globally from space using two instruments. The thermal infrared (TIR) and NIR sensor for carbon observation Fourier transform spectrometer (TANSO-FTS) detects gas absorption spectra in the solar shortwave infrared (SWIR) reflected on the Earth’s surface as well as of the thermal infrared radiated from the ground and the atmosphere. TANSO-FTS can detect NIR three narrow bands (760, 1600, and 2000 nm) and a wide TIR band (5500–14,300 nm) with a 0.2 cm^−1^ spectral resolution (interval). The TANSO cloud and aerosol imager (TANSO-CAI) is an ultraviolet (UV), visible, NIR, and SWIR radiometer designed to detect cloud and aerosol interference and to provide the data for their correction. GOSAT is placed in a sun-synchronous orbit at 13:00 local time, with an inclination angle of 98°.

GOSAT-2 was launched in orbit in 29 October 2018 as a successor to GOSAT (in orbit since 23 January 2009) [55]. Suto et al. [55] provided an overview of the TANSO-FTS-2 instrument, the level-1 processing, and the 1st-year in-orbit performance. GOSAT-2 monitors CO_2_ and CH_4_ in order to increase our understanding of the global carbon cycle and simultaneously measures CO emitted from fossil fuel combustion and biomass burning and permits identification of the amount of combustion-related carbon. To do this, the satellite utilizes TANSO-FTS-2, which can measure the oxygen A band (760 nm), weak and strong CO_2_ bands (1600 and 2000 nm), weak and strong CH_4_ bands (1600 and 2300 nm), a weak CO band (2300 nm), a mid-wave TIR band (5500–8400 nm), and a long-wave TIR band (8400–14,300 nm) with 0.2 cm^−1^ spectral sampling intervals. TANSO-FTS-2 is equipped with a solar diffuser target, a monochromatic light source, and a blackbody for spectral radiance calibration, allowing characterization of time-dependent instrument changes in orbit. The onboard-recalibrated instrumental parameters are considered in operational level-1 processing, and the TANSO-FTS-2 level-1 version 102102 products were officially released on 25 May 2020.

Imasu et al. [56] compared the GOSAT sensors and found that the sensors of GOSAT-2 offer higher performance in most respects. The quality and quantity of data from observations are expected to be improved accordingly. The signal-to-noise ratio (SNR) is better in both the SWIR and TIR bands of TANSO-FTS-2. This improvement ultimately enhances the accuracy of GHG concentration analysis. Furthermore, because of the improved SNR in the SWIR band, the northern limit at which data are obtainable in high-latitude regions of the Northern Hemisphere in winter, where observation data have remained unavailable because of weak signal strength, has moved to higher latitudes. As better data are obtained in greater quantities, progress in carbon cycle research for high-latitude regions is anticipated. Suto et al. [55] indicated that the spectral radiances measured by TANSO-FTS and TANSO-FTS-2 agree within 2 % of the averaged bias, with a 0.5 % standard deviation, for SWIR bands and that the agreement of brightness temperature between TANSO-FTS-2 and AIRS–IASI is better than 1 K in the range from 220 to 320 K. Suto et al. [55] confirmed that GOSAT-2 not only provides seamless global CO_2_ and CH_4_ observation but also observes local emissions and uptake with an additional CO channel, fully customized sampling patterns, higher signal-to-noise ratios, and wider pointing angles than GOSAT.

The resolutions of the vertical concentration distributions of CO_2_ and CH_4_ have been improved drastically [56]. The function introduced for GOSAT-2 that is not in GOSAT is an intelligent pointing mechanism: a cloud area avoidance function that uses the in-field camera of TANSO-FTS-2, which can increase the amounts of observation data globally and can improve the accuracy of CO_2_ emissions estimation and measurements of uptake intensity [56]. The effects are expected to be strong, especially for the tropics, because cumulus clouds are the most common cloud type and the intelligent pointing system can avoid the clouds effectively [56]. The other important benefit of TANSO-FTS-2 is that the wavelength range of Band 3 of SWIR has been expanded for measuring carbon monoxide (CO). CO originates from combustion, and it is used to evaluate some effects of human activities in urban areas and biomass burning in fields. In particular, black carbon-type aerosols can be measured by the sub-sensor, TANSO-CAI-2, to assess biomass burning along with CO_2_ and CO by TANSO-FTS-2.

The retrieval of GHG emissions from GOSAT and GOSAT-2 or any other satellite spectral data can be done using specific software. One capable example was proposed and investigated by Noël et al. [57], who showed new results from an updated version of the Fast atmOspheric traCe gAs retrievaL (FOCAL) retrieval method applied to measurements of GOSAT and GOSAT-2. FOCAL was originally developed for estimating the total column CO_2_ mixing ratio (XCO_2_) from spectral measurements made by the Orbiting Carbon Observatory-2 (OCO-2). However, depending on the available spectral windows, FOCAL also successfully retrieves total column amounts for other atmospheric species and their uncertainties within one single retrieval. The main focus of Noël et al. [57] is on methane (XCH_4_; full-physics and proxy product), water vapor (XH_2_O), and the relative ratio of semi-heavy water (HDO) to water vapor (δD). Due to the extended spectral range of GOSAT-2, it is also possible to derive information on carbon monoxide (XCO) and nitrous oxide (XN_2_O), the first published result of GOSAT-2 being a measurement of total column average XN_2_O.

The new FOCAL retrieval (v3.0) significantly increases the number of valid data for XCO_2_ compared with the previous FOCAL retrieval version (v1) by 50% for GOSAT and about a factor of 2 for GOSAT-2 due to relaxed pre-screening and improved post-processing [57]. All v3.0 FOCAL data products show a reasonable spatial distribution and temporal variations. Comparisons with the Total Carbon Column Observing Network (TCCON) result in station-to-station biases which are generally in line with the reported TCCON uncertainties [57].

Global XN_2_O maps show a gradient from the tropics to higher latitudes on the order of 15 ppb, which can be explained by variations in tropopause height [57]. The new GOSAT-2 XN_2_O product compares well with TCCON. Its station-to-station variability is lower than 2 ppb, which is about the magnitude of the typical N_2_O variations close to the surface [57]. However, both GOSAT-2 and TCCON measurements show that the seasonal variations in the total column average XN_2_O are on the order of 8 ppb peak-to-peak, which can be easily resolved by the GOSAT-2 FOCAL data [57]. Noting that only a few XN_2_O measurements from satellites exist so far, the GOSAT-2 FOCAL product will be a valuable contribution in this context [57].

CH_4_ is a key GHG with a strong climate impact and a relatively short atmospheric lifetime, making accurate monitoring essential for mitigation strategies [58]. Saito et al. [58] present an evaluation of the GOSAT-2 Level 4 (G2L4) CH_4_ flux product supported by analysis of the underlying Level 2 (L2) XCH_4_ retrievals and summarize key findings on global and regional CH_4_ budgets. Using an atmospheric inversion framework, Saito et al. [58] generated G2L4 posterior CH_4_ fluxes and assessed their consistency by comparing them with inversions constrained by alternative observational datasets, including GOSAT L2 retrievals and ground-based and aircraft measurements. GOSAT-2 achieved substantial improvements in observational coverage and data density compared to GOSAT, particularly in tropical and high-latitude regions. Posterior flux estimates derived from G2L4 are broadly consistent with global CH_4_ budgets reported in synthesis studies, while prior-to-posterior differences reveal positive corrections in tropical regions and negative adjustments in several mid-latitude industrial areas. A preliminary sector-focused assessment further demonstrates the potential of GOSAT-2 to inform anthropogenic CH_4_ emission evaluations in regions where such sources dominate. These findings highlight the capability of GOSAT-2 to refine regional and global CH_4_ emission estimates and underscore priorities for future improvements in retrieval algorithms, observation strategies, and integration with complementary datasets.

Schooling et al. [59] report on satellites that monitor changes in atmospheric GHG emissions, namely, the Japanese Greenhouse gases Observing SATellite (GOSAT) and the European TROPOspheric Monitoring Instrument (TROPOMI).

The details of GHG emission estimation from biomass burning using satellite-measured radiation interacting with atmospheric gases or satellite-derived data can be found in Kuze et al. [54], Suto et al. [55], Imasu et al. [56], Noël et al. [57], Saito et al. [58], Schooling et al. [59], and recent related publications.

## 2. GHG Emissions and GWP of Biomass Combustion Prediction by NIR Spectra Scanned on Intact Biomass Chips or Powder Calibrated Using HHV According to IPCC Guidelines

### 2.1. Relationship of Lignocellulosic Constituents and Elements in Biomass and HHV and GHG Emissions

The use of biomass energy can reduce GHG emissions, and it does not contribute to the risk of global climate changes as it does not increase CO_2_ emissions in the atmosphere [6]. Trees and plants remove carbon from the atmosphere through photosynthesis; therefore, if the amount of new biomass growth balances the biomass used for energy, bioenergy is CO_2_ neutral [6]. Biomass combustion stands as a renowned sustainable energy system that helps lower GHG release levels [60,61]. FAO [62] reported that agriculture contributes 17% of the world’s GHG emissions, with sugarcane plantations contributing approximately 11% of the total emissions from the agricultural sector [63]. Biomasses such as fast-growing trees, wood and non-wood, and agricultural residues are lignocellulosic biomasses whose main components are cellulose, hemicellulose and lignin, the major cell wall components of the fibers of all wood and agricultural waste [64,65,66], which have the formulas (C_6_H_10_O_5_)_n_, C_81_H_92_O_28_ and C_18_H_13_N_3_Na_2_O_8_S_2_, respectively [67].

**Figure 2:**
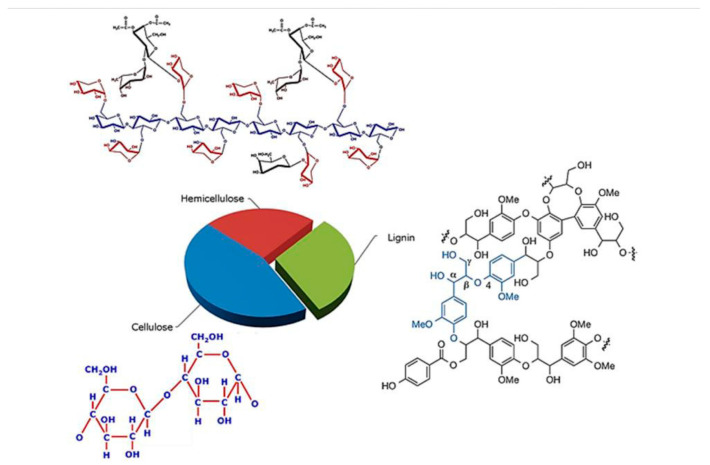
The chemical structures of cellulose, hemicellulose, and lignin [68].

Cellulose is a linear biopolymer comprised of D-glucose sugars linked by β-1,4 glycosidic bonds [6]. Hemicelluloses are polymers of various pentoses and hexoses, unlike cellulose, which has an amorphous structure and is composed of heterogeneous polysaccharides [6]. Lignin is composed of three common phenylpropane structures, including p-hydroxyphenyl, syringyl, and guaiacyl units [6]. Figure 2 shows the chemical structures of cellulose, hemicellulose, and lignin. Different kinds of hydrogen (H) bonds, including OH and CH bonds, were observed.

It is obvious according to the IPCC protocol that HHV is essential for GHG emission calculation. The elemental constitution of the biomass, including carbon (C), H, nitrogen (N), sulfur (S), and oxygen (O), measured by ultimate analysis, contributed in large part to the higher heating value (HHV) of the combustion as well as the related GHG output. Wood biomass C content significantly contributes to heat energy, H content improves combustion quality, a low N percentage is favorable for limiting NOx emissions, and a high O content, which is typical for biomass, decreases energy density [69]. By ultimate analysis, HHV = 0.2949C + 0.8250H, which was developed by Yin [70], who reported a mean absolute error (MAE) lower than 5% and a marginal mean bias error (MBE) of just 0.57%, which indicated that the H content contributed more to HHV compared to the C content. The low N and S contents in biomass dictate that the biomass cannot be an adverse source of environmental SOx and NOx [71]. Biomass combustion emits more NOx and N_2_O than coal and other fossil fuels at the same heating value [72].

C and H in high concentrations boost the HHV, making combustion more efficient and the CO_2_ output per unit of generated energy lower. Conversely, N and S in high concentrations release higher amounts of N_2_O and sulfur dioxide (SO_2_) and cause pollution of the environment. Higher concentrations of O and moisture (H_2_O), in turn, lower the HHV, resulting in incomplete combustion and a higher CH_4_ output. High C and H but low N, S, and H_2_O characterizes the best type of biomass for minimizing GHG output while maximizing the amount of energy, i.e., HHV.

HHV can normally be measured using an automatic bomb calorimeter, mainly according to the adiabatic principle and the isoperibol principle. The thermal dynamics of bomb calorimeters are modeled using a lumped heat transfer analysis in which heat is released in a pressure vessel/bomb immersed in a stirred water bath that is surrounded by a static air space bounded by an insulated (static) jacket, a constant/controlled temperature jacket (isoperibol), or a changing temperature (adiabatic) jacket [73]. The temperature history of the water bath for each of these boundary conditions (methods) is well described by the two-term solution for the calorimeter response to a heat impulse (combustion), allowing the heat transfer coefficients and thermal capacities of the bomb and water bath to be determined parametrically [73]. The validated heat transfer model provides an expression for direct calculation of the heat released in an arbitrary process inside a bomb calorimeter using the temperature history of the water bath for each of the boundary conditions (methods) [73].

Generally, a bomb calorimeter is calibrated with benzoic acid tablets which have a known and/or constant weight and a known and constant HHV. A cotton thread with a known and constant HHV is used for ignition in the bomb to measure the HHV of the grounded sample, which has previously been tabletized. The bomb calorimeter vessel for ignition is kept in a controlled-temperature environment, and during the burning of the biomass tablet, the environmental increase in temperature is recorded for HHV calculation. Including preparation, the total experimental time to measure HHV for each sample was approximately 40 min to 1 h.

### 2.2. Molecular Vibration of Hydrogen Bonds Caused by NIR Radiation Related to GHG Emissions

NIR spectroscopy has emerged as a promising tool for rapid and non-destructive analysis of biomass properties. NIR spectroscopy models can evaluate ultimate analysis data; for example, Posom and Sirisomboon [74] showed coefficient of determination (R^2^) of prediction set (R^2^P) values of 0.80 for C, 0.85 for H, and 0.97 for N for bamboo biomass. NIR hyperspectral images provide effective tools for the prediction of C, H, and N contents of commercial biomass pellets, including filter cake, *Leucaena leucocephala*, bamboo, cassava rhizome, bagasse, sugarcane leaves, straw, rice husk, eucalyptus bark, Napier grass, and corn cob pellets, developed using standard normal variate (SNV) spectral pretreatment and improved genetic algorithm (iGA) wavelength selection, providing the highest accuracy with an R^2^P and a standard error of prediction (SEP) of 0.83 and 1.33%, 0.84 and 0.17%, and 0.90 and 0.098% for C, H, and N contents, respectively [75]. Rice husk models for the HHV and lower heating value (LHV) provided root mean square errors of cross-validation (RMSECVs) of 119 J g^−1^ and 119 J g^−1^, respectively [76]. The HHV of maize cob could be evaluated [77] with an RMSECV of 91.1 J g^−1^. The HHV and LHV of ground cassava rhizome was investigated, where R^2^ values between 0.90 and 0.98 were obtained [78]. For ground bamboo, the HHV was predicted with an R^2^P of 0.92, a root mean square error of prediction (RMSEP) of 122 J g^−1^, a ratio of prediction to deviation (RPD) of 3.7, and a bias of 14.4 J g^−1^ [79]. The accuracy of the bamboo chip models was slightly lower than that of the ground bamboo studied by Posom and Sirisomboon [74,80]. The R^2^ values of bamboo chips and ground bamboo models for HHV and LHV were 0.84 and 0.92 and 0.89 and 0.93, respectively [80]. For most of these parameters, there was no statistically significant difference between the wood chips and the ground bamboo model.

It is evident that C, H, N and HHV, which are essential values for the calculation of GHG emissions, can be estimated by the rapid and non-destructive NIR spectroscopy method. However, Table 2 shows the methodological details of referenced models [74,75,76,77,78,79,80], including dataset size, calibration–validation strategy, preprocessing procedures, and hyperparameter selection with model performance metrics, and their applicability in the determination of the reliability and reproducibility of the referenced modeling frameworks.

**Figure 3:**
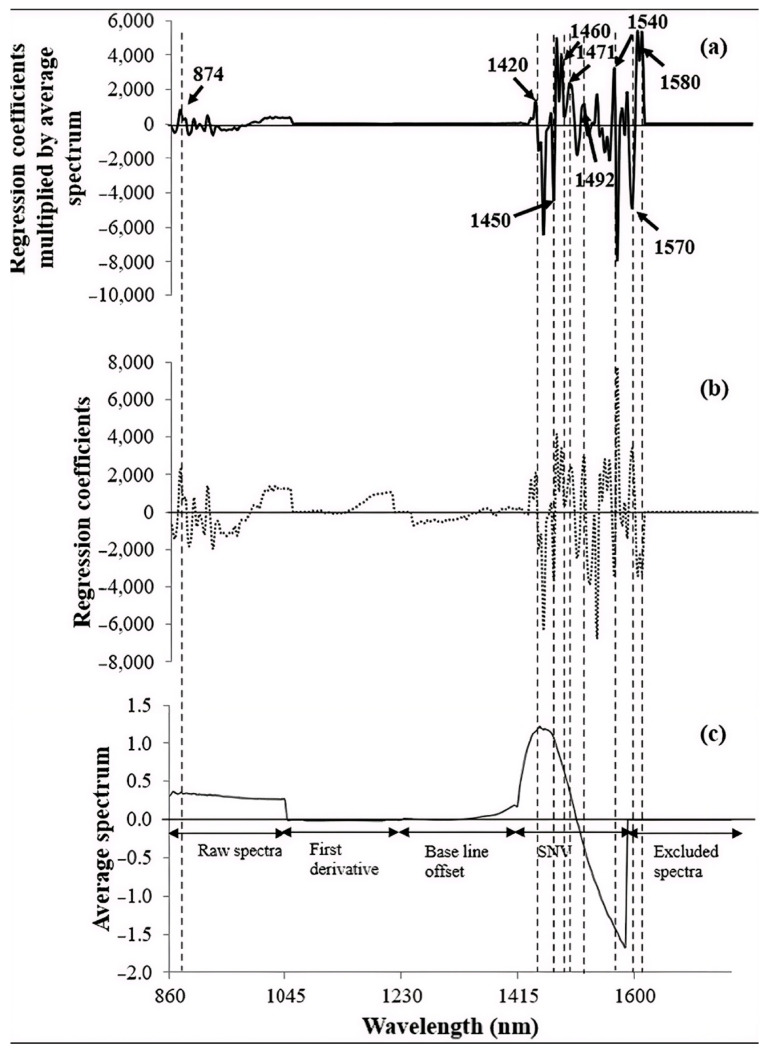
An example of a regression coefficient plot and an average pretreated spectrum of sugarcane bagasse for HHV prediction (**a**) The product spectrum of the absorption spectrum and the regression coefficient spectrum, (**b**) The regression coefficient spectrum, and (**c**) The average pretreated spectrum [89].

Due to the energy quality of biomass, HHV is a physical parameter which has no NIR absorption bands for direct correlation and can be predicted using the NIR model; therefore, it should be correlated with biomass chemical constituents with known absorption bands. The HHV of biomass is related to the lignocellulosic compound content, i.e., cellulose, hemicellulose and lignin [81,82,83], and, as was previously mentioned in Section 2.1 on the relationship of lignocellulosic compounds and elements in biomass and HHV and GHG emissions, there are NIR absorption bands at 1780, 1820, 2270, 2336, and 2488 nm for cellulose [84] and 1218, 1360, 1492, 1584, 1728, 1830, 2110, 2186, 2262, and 2314 nm for hemicelluloses [85]. For lignin, the wavelength range of 2449–1287 nm (4083–7773 cm^−1^) was used successfully for lignin prediction in corn stalks [86]. Jin and Chen [87], Liu and Chen [86], and Sabatier et al. [88] proved that NIR spectroscopy is suitable for the analysis of compounds (cellulose, hemicellulose and lignin) in lignocellulosic biomass. These lignocellulosic compound structures include C-H and O-H (Figure 2), which are NIR absorbers. Figure 3 shows NIR overtone and combination vibration of the NIR absorber in bamboo biomass, indicated by prediction model regression coefficient and X-loading values [67]. These show the agreement of the vibration of H bonds in lignocellulosic compounds in biomass with that of other compounds in biomass such as C-O in starch and C=O in amino acids. The peaks in the regression coefficient plots and X-loading plots of the optimized model can indicate that at the molecular level, for example, the highest peak at a wavelength in any direction indicates the highest positive or negative relationship between the molecular vibration with the attribute. The wavelength is the most effective indicator of prediction of an attribute in the optimized model for it can indicate the attribute most related bond vibration by the radiation.

Figure 3 shows an example of a regression coefficient plot and an average pretreated spectrum of sugarcane bagasse for HHV prediction. The peaks of the average pretreated spectrum and peaks in the regression coefficient plot and X-loading plot at the same wavelengths for an attribute prediction model, in our case GHG emissions and GWP of biomass combustion, can be used to infer the predicted quantity of the attribute based on the sum of the product of the absorption spectrum and the regression coefficient value at each wavelength throughout the whole spectrum (Figure 3a). High peaks in the regression coefficient plot (Figure 3b) and X-loading plot at the specific wavelengths of vibration of NIR absorbers contained in large amounts in the biomass i.e., cellulose, hemicellulose and lignin, can confirm the strong relationship between the NIR absorbers and the attributes, e.g., GHG emissions and GWP. Figure 3c is the average pretreated spectrum.

**Table 2:** The methodological details of the referenced models [74,75,76,77,78,79,80,89].

References	Biomass	Reference Parameter	Sample Size	Spectral Range	Preprocessing Method	Calibration–Validation Strategy	Model Algorithm	Hyperparameter	Performance Metrics (R^2^P, SEP/RMSEP, RPD, Bias)	Applicability (Refer to Table 3) ^^
[74] **Posom and Sirisomboon, 2017**	Ground and chipped bamboo	LHV C H N S	LHV: 80 C: 80 H: 79 N: 79 S: 78 O: 80	LHV: 8373.9–7853.2, 6827.2–5793.5 C: 12,493.3–11,602.3, 10,715.2–9824.2, 8937–7155, 6267.9–4485.9 H: 9403.8–7498.3, 5450.2–4597.7 N: 7857–6823.3, 5797.3–4246.7 S: 9403.8–7340.2, 6827.2–5276.6, 4763.6–4246.7 O: 8890.7–8370, 6310.3–5793.5	LHV: MSC C: 2nd derivative H: Constant offset elimination N: Min–max normalization S: MSC O: Straight-line substation	LHV: 64:16 C: 64:16 H: 63:16 N: 63:16 S: 62:16 O: 64:16	PLSR with external test set	Latent variables optimized by CV LHV: 3 C: 4 H: 9 N: 8 S: 8 O: 5	LHV: 0.934, 0.119 MJ kg^−1^, 4.0, −0.0187 MJ kg^−1^ C: 0.803, 0.532%, 2.3, −0.118% H: 0.856, 0.0427%, 2.7, −0.00524% N: 0.973, 0.0276%, 6.6, 0.0103% S: 0.785, 0.00761%, 2.2, −0.00139% O: 0.522, 1.110%, 1.5, −0.158%	LHV: **f** C: **d** H: **e** N: **f** S: **d** O: **c**
[75] **Pitak et al., 2021**	Different species of biomass pellets	C H N S	C: 160 H: 159 N: 159 S: 157	C: iGA selected 102 variables H: iGA selected 102 variables N: Full range 256 variables S: Full range 256 variables	C: SNV H: SNV N: SNV S: 2nd derivative	C: 116:44 H: 116:43 N: 115:44 S: 114:43	PLSR with external test set	Latent variables optimized by CV C: 6 H: 7 N: 7 S: 7	C: 0.83, 1.33%, 2.5, 3.3% H: 0.84, 0.17%, 2.8, 2.8% N: 0.91, 0.094%, 3.4, 13.2% S: 0.31, 0.026%, 1.3, 19.3%	C: **e** H: **e** N: **e** S: **b**
[76] **Nakawajana et al., 2018**	Ground rice husk	LHV HHV	65	LHV: 7425–5446.3 and 4601.6–4246.7 cm^−1^ HHV: 7425–5446.3 and 4601.6–4246.7 cm^−1^	LHV: MSC HHV: MSC	Calibration set: unknown set 50:15	PLSR with CV calibration and validated with unknown set	Latent variables optimized by CV LHV: 7 HHV: 7	LHV: 0.780, 119 J g^−1^, 2.1, −2.52 J g^−1^ HHV: 0.778, 119 J g^−1^, 2.1, −2.49 J g^−1^	LHV: **d** HHV: **d**
[77] **Posom and Nakawajana, 2018**	Milled maize cob	HHV	60	5450.2–4246.7 cm^−1^	2nd derivative	Calibration set: unknown set 50:10	PLSR with CV calibration and validated with unknown set	Latent variables optimized by CV HHV: 3	0.73, 91 J g^−1^, 1.9, 0.293 J g^−1^	
[78] **Nakawajana and Posom, 2019**	Ground cassava rhizome	LHV HHV	LHV: 49 HHV: 49	12,500- 3600 cm^−1^	PLS/SVM LHV: SNV/2nd derivative HHV:	Full CV LHV: 49 HHV: 49	PLSR with CV calibration	Latent variables optimized by CV for both PLSR and SVM LHV: 1 HHV: 1	PLSR LHV: 0.90, 241 J g^−1^, -, 0.224 J g^−1^, HHV: 0.90, 240 J g^−1^, -, 0.522 J g^−1^ SVM LHV: 0.85, 365 J g^−1^, -, −47.6 J g^−1^ HHV: 0.84, 364 J g^−1^, -, −47.2 J g^−1^	LHV: **e** HHV: **e**
[79] **Posom and Sirisomboon, 2017**	Ground bamboo	HHV	80	6102- 4597.7 cm^−1^	Min–max normalization	64:16	PLSR with external test set	Latent variables optimized by CV HHV: 7	0.92, 122 J g^−1^, 3.7 and 14.4 J g^−1^	**f**
[80] **Sirisomboon et al., 2020**	Bamboo chip	LHV HHV	LHV: 82 HHV: 83	LHV: 9403.8–5446.3, 4605.4–4242.9 cm^−1^ HHV: 8377.7–7853.2, 7344–6819.5 cm^−1^	LHV: 1st derivative + MSC HHV: Min–Max normalization	LHV 66:16 HHV 67:16	PLSR with external test set	Latent variables optimized by CV LHV: 5 HHV: 7	LHV 0.89, 0.12 MJ kg^−1^, 3.1, 0.016 MJ kg^−1^, HHV 0.84, 0.15 MJ kg^−1^, 2.5, 0.001 MJ kg^−1^	LHV: **e** HHV: **e**
[89] **Posom et al., 2022**	Sugarcane bagasse	HHV	100	860.6–1754.1 nm	Multiblock ^	75:25	PLSR with external test set	Latent variables optimized by CV HHV: 8	0.71, 206.6 J g^−1^, 1.9, −6.5 J g^−1^	

PLSR is partial least-squares regression, SVM is support vector machine, MSC is multiplicative scatter correction, CV is cross-validation, R^2^P is coefficient of determination for prediction set, SEP is standard error of prediction. RMSEP is root mean square of prediction, RPD is ratio of prediction to deviation, HHV is higher heating value, LHV is lower heating value, C is carbon, H is hydrogen, N is nitrogen, S is sulfur. ^ Multiblock 1. Raw spectra for Block 1 (860.6–1044.4 nm), 2 First derivative for Block 2 (1048.6–1229.1 nm), 3. Baseline offset for Block 3 (1233.2–1409.6 nm), 4 SNV for Block 4 (1413.5–1584.9 nm), 5. Excluded spectra for Block 5 (1588.7–1754.1 nm). ^^ Applicability of model is determined following Williams’ guidelines [Table 3].

These reviews indicate that it is possible to evaluate HHV and ultimate analysis parameters (C, H, N, O and S), which are derived from bomb calorimeters and element analyzers, respectively, for the evaluation of GHG emissions and GWP. However, to apply NIR spectroscopy, due to the broad band of vibration, chemometric methods (machine learning and or deep learning), such as partial least-squares regression (PLSR), support vector machines (SVMs), artificial neural networks (ANNs) and convolutional neural networks (CNNs), and so on, are needed in multivariate analysis to develop calibration models.

Table 2 shows the methodological details of an additional referenced model [89].

Table 3 shows an NIR spectroscopy application interpreted by Williams et al. [90] with an application example for biomass constituents, GHG emissions and GWP from biomass combustion or thermal conversion. The applications of the models in last column of Table 2 are indicated by symbols alongside the application work in Table 3. There are some factors which need to be taken into consideration, for example, the number of samples, for its robust use.

**Table 3:** NIR spectroscopy applications interpreted by Williams et al. [90] with application examples for GHG emissions and GWP from biomass combustion or thermal conversion.

R^2^ Range	Applicable Work	RPD—Biomass Constituents, Such as C, H, N, O, S, Cellulose, Hemicellulose, Lignin and GHG Emissions Measured Using Instrument	RPD—Biomass Functional Parameters Such as LHV, HHV, IPCC GHG Emissions, and IPCC GWP	Applicable Work ^
≤0.25	Cannot be used in NIR spectroscopy calibration	0.0 and 2.3	0.0–1.9	Its use is not recommended, **a**
0.26–0.49	Poor correlation and reason should be researched	2.4 and 3.0	2.0–2.4	Rough screening, **b**
0.50 and 0.64	Rough screening	3.1 and 4.9	2.5–2.9	Screening, **c**
0.66 and 0.81	Screening and some other “approximate” calibration	5.0 and 6.4	3.0–3.4	Quality control, **d**
0.83 and 0.90	Can be used with caution for most applications	6.5 and 8.0	3.5–4.0	Process control, **e**
0.92 and 0.96	Suitable for most applications	≥8.1	≥4.1	Any application, **f**
≥0.98	Excellent and usable for any application			**g**

**^ a**, **b**, **c**, **d**, **e**, **f**, and **g** is for referring in Table 2.

In addition, in Figure 3a, the spectrum of the product of the absorption spectrum and corresponding regression coefficients shows high peaks at 1420, 1450, 1460, 1492, 1540, 1570, and 1580 nm. NIR absorber vibration of major lignocellulosic compounds in biomass occurs at 1492 and 1584 (near the 1580 nm peak), which is the region associated with hemicellulose bands. The other band at 1450 nm is the water band; that at 1445 nm is the aromatic amine which is used to predict N; and that at 1583 nm is the alcohol or water band, which is used to predict O (Table 4). It can be noticed that these product peaks are in the range of 2449–1287 nm (4083–7773 cm^−1^), which is used for the effective prediction of lignin in corn stock [86].

**Figure 4:**
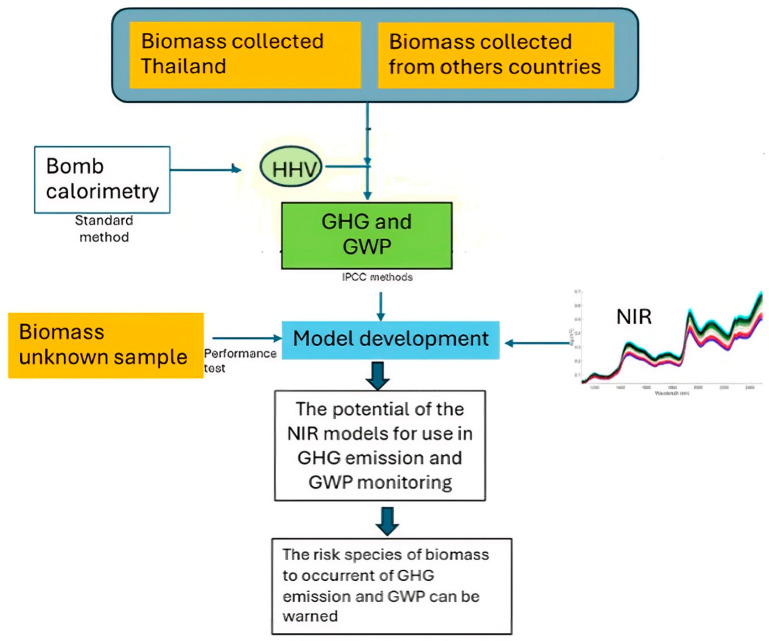
An example of an NIR spectroscopy procedure for evaluation of GHG emissions and GWP of biomass combustion.

Figure 4 shows the procedure of NIR spectroscopy for the evaluation of the GHG emissions and GWP of biomass combustion. The procedure started with the collection of biomass samples in Nepal and Thailand and other countries of different species at different times over a number of years to obtain robust models. The samples were subjected to NIR spectrum acquisition by an NIR spectrometer, and the samples were measured for HHV with a bomb calorimeter and subject to ultimate analysis by an elemental analyzer, and then calculations were performed for GHG emissions and GWP. Both datasets were subjected to prediction model development, including calibration and prediction, after which the unknown samples or independent sample set was subjected to the model to validate the prediction performance of the optimized model.

A description of model development and validation procedures

NIR (near-infrared) is infrared radiation in the wavelength range of 700–2500 nm, which is close to the visible range (VIS, 400–700 nm). Detection is typically performed using photodiode detectors such as germanium (Ge) and indium gallium arsenide (InGaAs) and some others such as lead sulfide (PbS) and silicon (Si). NIR shortwave is in the shortwave region of 700–1100 nm. NIR longwave is in the wavelength region of 1100–2500 nm. Organic matter can well absorb NIR as it has hydrogen bonds in its molecules. The NIR spectrum is characterized by broad bands and overlapping among some molecular bonds, making it impossible to make direct quantitative interpretations like those in the mid-IR (MIR) range. Chemometrics are therefore necessary to make use of the spectrum. Chemometrics can correlate the absorption or reflection or transmission variables (independent variables) with parameters of interest (dependent variables), e.g., HHV, C, H, N, O, and S, in biomass samples and the GHG emissions and GWP of biomass combustion. In this review, some chemometric modeling algorithms were used, including partial least squares regression (PLSR) and support vector machines (SVMs). These kinds of models can deal with full-wavelength-range spectrum variables which exhibit multicollinearity, some non-informative variables with respect to dependent variables, and the physical properties of samples and scanning environment effects. The way to remove these adverse effects is by spectral pretreatment and selection of the feature variable (wavelength). The spectral pretreatment methods used in our review include the standard normal variate and second-derivative algorithm and feature variable selection by iGA and iSPA. Modeling involves calibration model development and developed model validation. The samples collected have to be divided into a calibration set and a prediction set with the same distribution for no bias (fair) to indicate the developed model’s performance. This requires an algorithm for separation of the sample dataset, of which there are several, for example, the commonly used Kennard–Stone (KS) algorithm. However, to prove the robustness or the applicability of the model, an independent sample set or an unknown sample set which is within the range of the calibration set is necessary.

The knowledge on chemometrics in spectral analysis and modeling can be read in Chu et al. [91]. Some explanations related to this review in Section 2.2 are given in following.

First- and second-derivative spectrum pretreatment are commonly used preprocessing methods for baseline correction and resolution enhancement in spectral analysis, generally applied in two types of methods for spectral derivation: direct difference and derivative methods [91]. The direct difference (Norris) method is the simplest derivative method for discrete spectra. Savitzky–Golay (S-G) convolution smoothing and the wavelet transform can also be used to obtain spectral derivatives. The standard normal variate (SNV) method is primarily used to eliminate the effects of solid particle size, surface scattering, and optical path changes on NIR diffuse reflection spectra [91,92].

Kennard–Stone (KS) is a method for dividing samples into calibration (training) and prediction (test/validation) sets. The algorithm ensures that the variability of each subset represents the variability of the entire dataset. It selects samples based on maximum Euclidean distance (similar to ruler distance calculated across spectral wavelengths). The first two samples are those farthest apart, and subsequent samples are selected iteratively based on the maximum distance from already selected samples using a stepwise procedure.

The featured wavelength or characteristic wavelength is a wavelength that provides highly relevant information about the dependent variable with minimal noise. Using selected wavelengths instead of the full spectrum improves accuracy and precision, computational efficiency (smaller size of data), and computer cost and size. It also enables the development of multispectral imaging systems.

A genetic algorithm (GA) is an optimization algorithm inspired by natural selection and evolution used to identify characteristic wavelengths. The main components are encoding variables, an initial population (chromosomes and genes), fitness evaluation, crossover (reproduction), and mutation.

A GA searches for optimal solutions through iterative evolution and can identify multiple optimal wavelengths simultaneously.

The successive projection algorithm (SPA) of without variable elimination, is an algorithm used to select characteristic wavelengths by minimizing collinearity by defining parameters (number of wavelengths, spectral angle threshold, and spatial threshold), selecting the first wavelength based on the maximum pixel intensity, selecting the second wavelength based on the maximum distance from the first, applying orthogonal projection to identify additional wavelengths, repeating the process until the required number of wavelengths is obtained, determining the optimal number of wavelengths using RMSEP and optional elimination of irrelevant variables using a relevance index and statistical testing (f-test).

SPA elimination with variable elimination is a successive projection algorithm enhanced with an additional variable elimination step to improve model parsimony.

Partial least squares regression (PLSR) is an algorithm for developing regression models relating spectral data (independent variables) to dependent or response variables. It reduces dimensionality using principal component analysis (PCA) and then constructs regression equations using multiple linear regression or regression coefficients to predict dependent variables. GA-PLSR and SPA-PLSR are modeling approaches in which PLSR uses wavelengths selected by GA and SPA as input variables.

The support vector machine (SVM) is a machine learning algorithm used for regression, classification and clustering. The SVM identifies an optimal hyperplane that maximizes the margin between classes. It uses kernel functions to transform non-linear data into a higher-dimensional space for better separation.

The iSPA (interval successive projection algorithm) and iGA (interval genetic algorithm) are different from the GA and SPA. The SPA selects individual wavelengths (single variables) one by one from the full spectrum. Its aim is usually to reduce collinearity and find a small set of informative wavelengths, i.e., a set of discrete wavelengths. It is very parsimonious, simple, and useful for multispectral designs, though it has the limitation that it may miss useful neighboring spectral information. The iSPA selects intervals (spectral regions) rather than only single wavelengths and works with groups of adjacent wavelengths, providing one or more informative spectral bands/regions. It preserves local spectral structure and band shape, but may include some non-informative wavelengths inside the selected interval. The iGA applies the GA idea to spectral interval selection instead of single wavelengths. So, optimization is performed on spectral regions. Chromosome representation, where genes often encode interval start/end positions or interval masks, provides one or more selected wavelength regions.

Interval methods such as the iSPA and iGA are often preferred when useful chemical information is distributed across a band rather than exactly at one wavelength. This is important in NIR, as NIR bands are usually broad and overlapping, and neighboring wavelengths often carry related information, such that selecting an interval can be more chemically meaningful.

### 2.3. Sugarcane Bagasse’s and Other Biomass Species’ GHG Emissions and GWP

Japan supported biomass energy projects in emerging Southeast Asian countries (Thailand, Indonesia, Viet Nam, Myanmar, Philippines, Malaysia, Cambodia, and Laos), all of whose projects included boiler, turbine, and generator (BTG) technologies for direct combustion, co-firing, and incineration [93], indicating the importance of these technologies. The highest amounts of BTG technology [93] were in observed in Viet Nam, Myanmar and Malaysia, though they have different economic classifications, i.e., they are a developing country with a lower-middle-income economy; a least developed country (LDC); and a developing, upper-middle-income country, respectively. These factors indicate that BTG is a handleable technology independently of the economic status of the country in question.

The GWP of biomasses, including wood chips of fast-growing trees and agricultural waste, reported by Gyawali et al. [20], was calculated using the IPCC method and predicted by NIRS using a PLS regression model with the first-derivative spectra of the 325 wavenumbers obtained by the covariance matrix (COVM) variable selection method used as input, which outperformed the other predictive model’s performance regarding GWP, providing R^2^_C_ and R^2^_P_ values of 0.92 and 0.85, indicating a linear relationship between the predicted and calculated values during both the calibration and prediction, respectively; the RMSEC of 0.00053 kg CO_2_eq and the RMSEP of 0.00063 kg CO_2_eq show low prediction errors, and the bias (average error) of −0.00014 kg CO_2_eq and the RPD of 2.6 signify the fair predictive capability of the model for functional parameters, including GWP, as values of 2.5–2.9 were considered for screening applications indicated by Williams’ guidelines [90] in Table 3. These high R^2^_P_ values (0.85) in predicting GWP indicate that the model is usable with caution for most applications, including research, where the threshold of R^2^ indicated by Williams’ guidelines is between 0.83 and 0.90 [90] in Table 3.

Shrestha et al. [94] evaluated HHV for fast-growing trees and agricultural residues, including sugarcane bagasse, using Fourier transform near-infrared spectroscopy (FT-NIRS), scanning in diffused reflection mode at wavenumbers of 10,000–4000 cm^−1^ (1000–2500 nm), where 200 samples were collected. The models were developed using the correlation selected variable method + the PLS algorithm along with standard normal variate and first-derivative pretreatment, yielding a highly effective model exhibiting an R^2^_P_ of 0.88 and an RMSEP of 278.0 J g^−1^.

Sugarcane bagasse was selected as a sample for the case study as it was proved through comprehensive analysis to have significant potential for thermochemical conversion systems and to be important in selecting and designing fluidized bed technologies like pneumatic conveying, drying, combustion, and gasification equipment [95]. Using 150 kg of sugarcane bagasse collected from the Serra mill in the central region of São Paulo gathered from stored stacks located in the mill’s backyard, referred to as “bagasse in nature” or raw bagasse, consisting of a diverse range of polydisperse particles of varying sizes and shapes, a mixture of various sugarcane varieties from different geographical locations, soil types, harvest times, weather conditions, and harvest forms (manual and mechanized), Pérez et al. [95] analyzed the particle size distribution with a mean geometric diameter of 0.722 mm as well as physical and chemical properties, including density measurements and HHV, and performed thermogravimetric analysis (TGA/DTA) and compositional, proximate, ultimate, and CHNS and O analysis. The result shows that the raw bagasse had higher volatile matter, fixed carbon, and ash contents and an HHV of 16 MJ kg^−1^, with a lower moisture content (8.71%), and thermal analysis indicated a peak degradation temperature for organic matter at 310–330 °C. The bagasse exhibited a higher combustion index than fossil fuels and other biomasses [95]. Logarithmic models were obtained to determine the real, particle, and apparent densities of bagasse, with the mean particle size within the 0.075–9.5 mm range, showing adequate results for particles with a mean diameter greater than 0.15 mm [95].

Sugarcane bagasse has attracted growing global interest due to its promising applications in renewable energy systems, including its use in bioenergy conversion processes and biofuel generation, as well as its use as a precursor for developing catalysts and advanced materials; furthermore, the importance of sugarcane bagasse valorization in mitigating GHG emissions, promoting circular economy practices, and accelerating the global transition toward sustainable and low-carbon energy systems has been highlighted [96], as discussed by da Silva Aires et al. [96] in a comprehensive bibliometric analysis of international research trends from 2005 to 2024 using data from 26,663 documents retrieved from the Web of Science database, ultimately refined to 657 relevant publications, which identified Brazil, India, the United States, and China as the leading contributors in this field, reflecting their strategic investments in biomass research, energy policy, and technological development. The sugarcane industry mainly uses bagasse to produce electricity in sugar and ethanol plants, reducing GHG emissions by replacing fossil fuels with renewable biomass [96,97]. Brazil, the world’s largest producer of sugarcane, accounts for approximately 40% of global production.

Electricity generated from bagasse and straw has the potential to substantially mitigate GHG emissions and has the capacity to reduce emissions from the electricity sector by up to 13%, potentially avoiding up to 72 million tons of CO_2_ eq per year [96,98]. The sugar industry produces large quantities of bagasse, which is often underutilized and represents the most abundant agricultural waste [96,99]. The main challenge developing countries face is the accumulation of organic waste [96,100]. Energy production from sugarcane bagasse presents a promising solution to this problem [96,101]. By harnessing this waste for energy, an integrated approach can be adopted that promotes sustainability and aligns directly with Sustainable Development Goals (SDGs) 7 and 12 [96,102].

**Figure 5:**
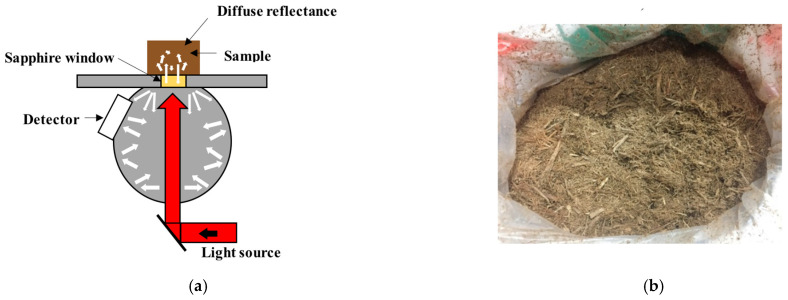
(**a**) Diagram of FT-NIR scanning in diffuse reflectance mode and (**b**) fresh bagasse collected from sugarcane mill.

**Figure 6:**
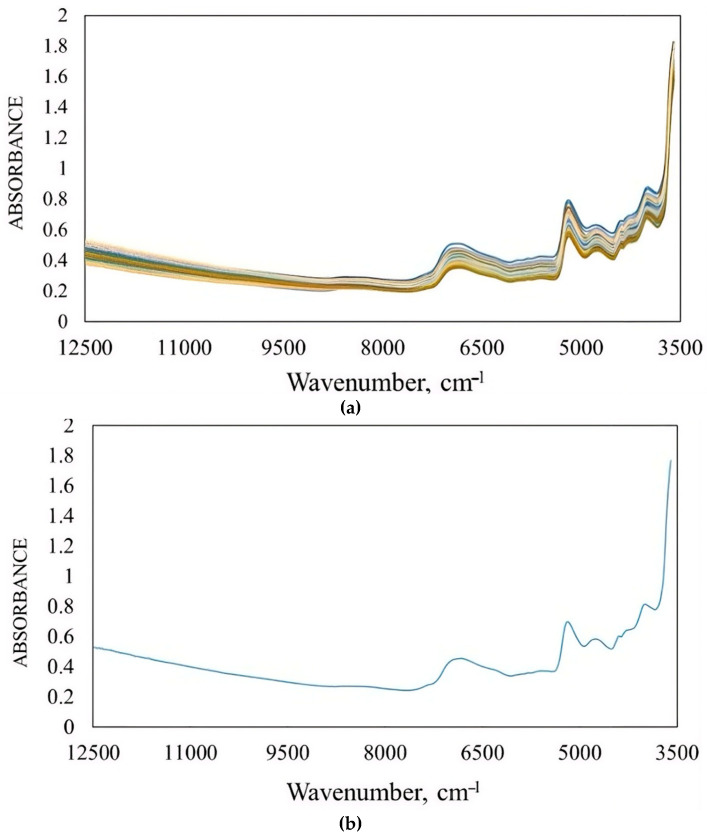
NIR spectra of sugarcane bagasse: (**a**) the spectra of 56 sugarcane bagasse samples and (**b**) the spectrum of a sugarcane bagasse sample collected from Khon Kaen Sugar Power Plant Co., Ltd., Factory 1, in Nam Phong District, Khon Kaen, Thailand.

This case study presents the application of an in-house NIR spectroscopic model developed in the near-infrared spectroscopy research center for agricultural product and food (www.nirsresearch.com)(accessed on 2 December 2025) at King Mongkut’s Institute of Technology Ladkrabang (KMITL), Bangkok, Thailand. The objective was to predict GHG emissions and the corresponding GWP resulting from the combustion of sugarcane bagasse sample collected from the Khon Kaen Sugar Power Plant Co., Ltd., Factory 1, in Nam Phong District, Khon Kaen, Thailand. NIR spectra were acquired in the 12,500–4000 cm^−1^ range (800–2500 nm), selected for its main strong absorption bands (8000–4000 cm^−1^) which correspond to the vibration of C-H, O-H, and N-H molecular bonds. Figure 5 shows (a) diagram of FT-NIR scanning in diffuse reflectance mode and (b) fresh bagasse collected from a sugarcane mill. Figure 6 shows (a) the spectra of 56 sugarcane bagasse samples and (b) the spectrum of a sugarcane bagasse sample collected from Khon Kaen Sugar Power Plant Co., Ltd., Factory 1, in Nam Phong District, Khon Kaen, Thailand, with an HHV value of 15,069.5 kJ kg^−1^ (0.0000150695 TJ kg^−1^) measured by a bomb calorimeter and 15,311.0 kJ kg^−1^ (0.0000153110 TJ kg^−1^) predicted by the NIR spectroscopic model developed in-house.

Spectral analysis and multivariate calibration enabled the quantification of emissions in units of kg TJ^−1^ for each gas. The gas emissions predicted by the NIR spectroscopic model are shown in Table 5.

Table 5 shows the comparison of the GHG (CO_2_, CH_4_, and N_2_O) emissions and GWP of sugarcane bagasse samples included in this case study calculated according to the IPCC guidelines (AR6) with the values obtained by NIR model prediction for HHV and GWP. The reference measured for HHV was obtained using a bomb calorimeter, and the reference calculated for GWP was calculated based on the GWP and HHV relationship, which is linear. The equation is expressed [20] as GWP or GWP total  =  2098.0 ×  HHV in TJ kg^−1^  =  0.000002098  ×  HHV in kJ kg^−1^  =  0.000002098  ×  HHV in J g^−1^. This make the GWP predicted by the HHV-PLSR model and the GWP predicted by the GWP-PLSR model equal.

The EFs for the calculation of GHG emissions were based on biomass combustion standards from IPCC guidelines: 112 kg CO_2_, 30 kg CH_4_, and 4 kg N_2_O per TJ of kg fuel burnt. The gas emissions were calculated using the IPCC calculation methods using the HHV of the bagasse measured with a bomb calorimeter by means of Equation (1).

CO_2_ Emissions (kg) = 1 kg × 0.0000150695 TJ kg^−1^ × 112 kg TJ^−1^ = 0.001687784 kg

CH_4_ Emissions (kg) = 1 kg × 0.0000150695 TJ kg^−1^ × 30 kg TJ^−1^ = 0.000452085 kg

N_2_O Emissions (kg) = 1 kg × 0.0000150695 TJ kg^−1^ × 4 kg TJ^−1^ = 0.000060278 kg

The HHV predicted by the NIR spectroscopic model is 15,311.0 kJ kg^−1^ using the NIR spectrum of sugarcane bagasse shown in Figure 6b. The gas emissions predicted based on the HHV value by the NIR spectroscopic model were: CO_2_: 0.001714832 kg TJ^−1^; CH_4_: 0.00045933 kg TJ^−1^; and N_2_O: 0.000061244 kg TJ^−1^. The values were calculated by means of Equation (1).

CO_2_ Emissions (kg) = 1 kg × 0.0000153110 TJ kg^−1^ × 112 kg TJ^−1^ = 0.001714832 kg

CH_4_ Emissions (kg) = 1 kg × 0.0000153110 TJ kg^−1^ × 30 kg TJ^−1^ = 0.000459330 kg

N_2_O Emissions (kg) = 1 kg × 0.0000153110 TJ kg^−1^ × 4 kg TJ^−1^ = 0.000061244 kg

Using Equation (2), the GWP of the sugarcane bagasse samples was calculated by means of the IPCC method using the GHG emissions calculated based on the HHV measured by a bomb calorimeter and predicted by NIR spectroscopy as follows:

GWP total (calculated using GHG emission calculated by using HHV measured by bomb calorimeter)

= (1 × 0.001687784) + (29.8 × 0.000452085) + (273 × 0.000060278)

= 0.001687784 + 0.013472133 + 0.016455894

= 0.031615811 kg CO_2_eq

GWP total (predicted by NIR spectroscopy)

= (1 × 0.001714832) + (29.8 × 0.000459330) + (273 × 0.000061244)

= 0.001714832 + 0.013688034 + 0.016719612

= 0.032122478 kg CO_2_eq

The EF of the biomass was calculated in the unit of kg CO_2_eq per metric ton of biomass burned using Equation (6):

EF of biomass (kg CO_2_eq (metric ton) ^−1^ of fuel burned) by IPCC calculation

= 0.031615811 × 1000 kg (metric ton) of fuel burned

= 31.62 kg CO_2_eq (metric ton) ^−1^ of fuel burned

EF of biomass (kg CO_2_eq (metric ton) ^−1^ of fuel burned) predicted by NIR model

= 0.032122478 × 1000 kg (metric ton) of fuel burned

= 32.12 kg CO_2_eq (metric ton) ^−1^ of fuel burned

Due to sugarcane bagasse being a carbon-neutral biomass, the EF of CO_2_ is deducted and the effective EF of sugarcane bagasse is equal to 29.93 kg CO_2_eq (metric ton) ^−1^ of fuel burned (by IPCC calculation) and 30.41 kg CO_2_eq (metric ton) ^−1^ of fuel burned according to NIR model prediction.

The CC of 1 kg of bagasse can be calculated by means of Equation (5), where the CC of sugarcane bagasse is 45.5% [103] and the HHV is 15,069.5 kJ kg^−1^ bagasse (0.0000150695 TJ kg^−1^ bagasse):

CC = 0.455 × 1 kg bagasse/0.0000150695 TJ kg^−1^ bagasse

= 30,193.437 kg TJ^−1^

The EF of gross CO_2_ determined by complete combustion of sugarcane bagasse can be calculated using Equation (4):

EF of CO_2 gross_ = 1 × 30,193.437 × 1 × (44/12) = 110,709.269 kg TJ^−1^

Table 6 shows the emission factors (EFs) and CO_2_eq (kg CO_2_eq ton^−1^ bagasse) for sugarcane bagasse-fired boilers in Brazil [104] compared to the values obtained for sugarcane the bagasse samples in this case study by means of the NIR spectroscopic model for emission of bagasse combustion.

The CO_2_eq (kg CO_2_eq [ton Bagasse]^−1^) of sugarcane bagasse-fired boilers in Brazil [96] was nearly 2-fold lower than that of the sugarcane bagasse in our sample, mainly due to the fact that it was calculated based on the lower heating value of bagasse [107] of 7536 MJ ton^−1^ (7.536 × 10^−6^ TJ kg^−1^), where the bagasse was considered to have a zero moisture content, while the heating value of bagasse in our calculation (15.0695 × 10^−6^ TJ kg^−1^) was HHV (the total amount of energy obtained by a unit of biomass completely burnt in a bomb calorimeter without deduction of the energy obtained by heating of vapor (biomass vapor evaporated during biomass burning) condensation) [47].

This result demonstrates the feasibility and practical accuracy of NIR spectroscopy for determining the HHV and GWP of sugarcane bagasse, particularly using localized, in-house models [20,94], for a time saving of at least 40 min per samples and non-destructive GHG emission, GWP and EF prediction. However, the prediction will be much faster if a specific model is generated separately for each GHG and the total GHG emissions. The study also highlights the potential of deploying the techniques in biomass-rich regions, for example, Nepal and Thailand, to support environmental monitoring, emissions reporting, and sustainable agricultural residue utilization.

Table 7 shows the HHV of different species of biomass measured in some research and GHG emissions calculated using the following equations:

CO_2_ emission (kg [kg Biomass] ^−1^) = HHV × 10^−6^ (MJ kg^−1^) × 112

CH_4_ emission (kg [kg Biomass]^−1^) = HHV × 10^−6^ (MJ kg^−1^) × 30

N_2_O emission (kg [kg Biomass]^−1^) = HHV × 10^−6^ (MJ kg^−1^) × 4

and IPCC-based GWP calculated [20] using

IPCC-based GWP (kg CO_2_e) = 0.002098 × HHV (MJ kg^−1^)

The linear dependency between HHV and IPCC-based GWP demonstrates that rapid spectroscopic prediction of HHV can serve as a reliable proxy for estimating GHG emissions from biomass combustion.

**Figure 7:**
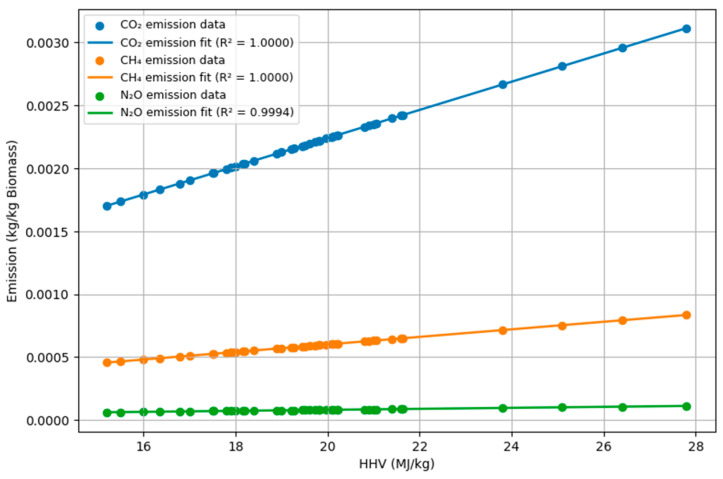
Relationship between measured HHV data in Table 7 and corresponding GHG emissions.

Figure 7 shows the relationship between HHV and GHG emissions in the biomasses listed in Table 7, indicating the linearity of the relationship due to the IPCC principle of calculation according to which more heat energy in biomass causes higher emissions, i.e., global heat. The graph shows that the rate of CO_2_ increase is highest, with the largest amount of gas emissions, followed by CH_4_ and N_2_O, respectively. A similar graph for HHV and GWP is shown in Gyawali et al. [20].

### 2.4. Comparative Advantages and Limitations of NIR Spectroscopy with Respect to Other Methods

NIR spectroscopy proposed compared to UAV and satellite systems

GHG emission and GWP estimation by destructive evaluation of the HHV of biomass according to the IPCC guidelines is well established and used worldwide. However, direct integration into a single non-destructive predictive framework linking NIR spectra of intact biomass before combustion to IPCC-based GHG emissions and GWP is not yet widely reported, which is only one report of our group [20], and as such it can reasonably be positioned as novel in terms of methodology and application. The paradigm shift represented by our proposed method, NIR spectroscopy for estimation of GHG emissions and GWP, allows estimation of what will be emitted (and how much) from a biomass sample determined on globe. While current UAV and satellite systems provide top-down estimates of biomass burning emissions based on atmospheric plume observations at a regional or global scale, our fuel-based methodology predicts emission potential directly from the intrinsic chemical composition of biomass by introducing a bottom-up, non-destructive NIR spectroscopic methodology to predict GHG emissions and GWP from biomass prior to combustion, offering a complementary and potentially integrative framework for multi-scale carbon monitoring.

NIR spectroscopy proposed compared to field biomass burning experiments

Field measurement captures what actually happens, including all real effects such as combustion inefficiency and environmental variability, whereas NIR spectroscopy captures what a biomass is and predicts emissions based on chemical composition and energy content. Field biomass burning experiments provide realistic emission profiles by capturing the full complexity of combustion dynamics under natural conditions. It should be noted that NIR-based estimation cannot inherently account for combustion-phase variables such as oxygen availability, flame temperature, and turbulence, which significantly influence CH_4_ and N_2_O emissions in field conditions. However, field experiment methods are costly and time-consuming, and if biomass species are heterogeneous, they require big areas to collect samples and are unsuitable for rapid screening or pre-combustion decision-making. Regarding GHG emission measurement accuracy and precision, normally the values are estimated by measurement of the ash quantity in pre-fire and post-fire biomass through a gravimetric method for drying and incineration, and the corresponding carbon content (in %) is calculated by multiplying the % biomass without ash by 0.58, which is the content of carbon in dry organic matter. This requires a number of samples for an experiment for acceptable accuracy and precision. In contrast, NIR spectroscopy enables fast, non-destructive prediction of biomass properties governing GHG formation, offering a scalable approach for estimating emissions prior to combustion through chemometric modeling, though the accuracy is dependent on the reference method for developing calibration models. Field measurement and NIR spectroscopy are complementary, not in competition: field experiments are needed for ground truth (validation and EF development) and NIR spectroscopy is a predictive tool (screening, optimization, and AI integration).

NIR spectroscopy proposed compared to laboratory biomass direct combustion-based experiments

Direct methods measure emissions after combustion and NIR spectroscopy predicts emissions before combustion via material properties, and these measurements have key conceptual differences. NIR spectroscopy does not measure GHGs directly but predicts them via the organic structure (C–H, O–H, and N–H bond vibration) of the lignocellulosic composition calibrated with ultimate analysis (C, H, N, O, and S) and HHV. These are fundamental drivers of CO_2_, CH_4_, and N_2_O formation. Direct combustion-based measurement methods are time-consuming, destructive, and unsuitable for rapid screening.

The accuracy and precision of GHG emissions quantification depend strongly on the analytical instruments employed. Higher accuracy is generally achieved when using high-performance or advanced measurement technologies. High-end systems provide reference-quality data with superior accuracy and sensitivity, include ultra-high-precision laser-based instruments, such as off axis integrated cavity output spectroscopy (OA-ICOS) analyzers, cavity ring-down spectroscopy (CRDS), and tunable diode laser absorption spectroscopy (TDLAS), which offer excellent sensitivity and real-time detection capabilities. Gas chromatography (GC)-based systems, including GC-FID, GC-ECD, and GC-TCD, are widely regarded as reference methods due to their high accuracy and reliability. In addition, Fourier transform infrared (FTIR) gas analyzers, both portable emission analyzers and laboratory-based systems, which enable simultaneous multi-gas detection, are commonly applied in combustion studies. Continuous emission monitoring systems (CEMS) are extensively used in industrial applications for stable, continuous, and long-term measurements. Non-dispersive infrared (NDIR) and electrochemical gas analyzers represent standard technologies, with NDIR being the most commonly used method for CO_2_ and CH_4_ detection due to its robustness and cost-effectiveness. Furthermore, emerging and frontier technologies, such as dual-comb spectroscopy, quantum cascade laser (QCL) systems, frequency comb spectroscopy, and advanced multi-gas analyzers (e.g., MIRO Analytical systems), provide cutting-edge capabilities with ultra-high resolution and sensitivity, although they are currently limited mainly to advanced research applications.

However, cost-effective alternatives, including portable non-dispersive infrared (NDIR) and Fourier transform infrared (FTIR) analyzers, offer practical solutions for field applications. Low-cost instruments are suitable for screening, preliminary studies, educational purposes, and field surveys with limited budgets. These include NDIR CO_2_ sensors (e.g., SenseAir and Vaisala entry-level models), low-cost CH_4_ sensors (e.g., Figaro Engineering Inc. MQ-series), and electrochemical gas sensors for CO and NOₓ detection. Medium-cost instruments are more appropriate for academic research, pilot-scale studies, field biomass burning experiments, and routine monitoring. These include portable multi-gas NDIR analyzers, electrochemical multi-gas analyzers, and entry-level portable FTIR systems.

Advantages of NIR spectroscopy proposed (pre-combustion prediction)

The advantages of NIR spectroscopy include its not requiring combustion (making it safe and environmentally friendly), its non-destructive and rapid nature (direct scanning of biomass chips/powder before burning) taking only seconds per sample, its high throughput (hundreds of samples day^−1^), and even inline or online scanning over or under or on the side of the biomass conveying machine, as well as its field deployability (portable NIR spectrometer), low operational cost after calibration, and prediction of multiple parameters simultaneously (CO_2_ emission, CH_4_ emission, N_2_O emission, gross GHG emission and GWP) via modeling without long experiments or calculations, enabling real-time decision-making (feedstock selection before burning) and strong AI integration.

Disadvantages of NIR spectroscopy proposed (pre-combustion prediction)

NIR spectroscopy is an indirect model-based method (requiring calibration with reference methods) that is dependent on dataset quality (sample diversity and representativeness are critical for model development), characterized by spectral complexity (broad overlapping bands of overtones/combinations vibration are weak compared to fundamental vibration by mid IR radiation), and its absolute accuracy and comparability with respect to direct measurements are limited, especially for trace gases (CH_4_ and N_2_O), in addition to model transferability issues (different biomass types and instruments) and the fact that it needs advanced preprocessing of spectra (SNV, MSC, derivatives, and wavelength selection) to eliminate biomass sample physical effects and environmental noise.

## 3. Conclusions

In this review, the possibility of using NIR spectroscopy to evaluate major GHG emissions from biomass combustion, including CO_2_, CH_4_, and N_2_O, as well as GWP and EFs, is confirmed, showing it to be an alternative to IPCC methods for the estimation of these climate change-related factors, highlighting it as a very rapid non-destructive method which can scan the spectra of a biomass sample without any pretreatment process in about 10 s, with no chemicals used, no expert needed, and no sophisticated procedure, at a very low operation cost and in a short time, all of which are strong selling points. NIR spectroscopy is a novel method for predicting GHG emissions and GWP directly from intact chip or powder biomass spectral data without explicit gas measurement.

The method described in this paper is a simple protocol which can be used to measure how much the global temperature has risen due to combustion processes through the determination of the GHG emissions and GWP of biomass combustion, contributing to consideration of the mean reduction in global temperature rise and fostering a transition towards more sustainable energy systems.

## Figures and Tables

**Table 1:** Key factors affecting carbon neutrality of biomass combustion.

Factor [References]	Mechanism	Governing Equation
Supply chain [36,37]	Harvesting, transport, processing emissions	Emission_total = Emission_combustion + Emission_supply_chain
Carbon debt [38,39]	Time lag between emission and regrowth	Carbon debt = Emission_initial − Emission_uptake(t)
Land-use change (LUC) [39,40]	Loss of biomass + soil carbon	ΔC = C_before − C_after
Combustion inefficiency [41]	Incomplete combustion → CH_4_, N_2_O	GWP = CO_2_ + 29.8 CH_4_ + 273 N_2_O

**Table 4:** NIR overtone and combination vibration of NIR absorber in Bamboo indicated by prediction model regression coefficient and X-loading [67,74].

Content Prediction	Wavenumber (cm^−1^) (nm)	Prediction Model Regression Coefficient and X-Loading
C	12331 (810 nm)	808 nm corresponded to 2 × N–H stretching + 2 × N–H deformation + 2 × C–H stretching
	5273 (1896 nm)	1900 nm corresponded to O–H stretching + 2 × C–O stretching of starch; X-loading peaks observed at ~5331 cm^−1^ (1876 nm)
	5253 (1903 nm)	1900 nm corresponded to O–H stretching + 2 × C=O stretching of starch
	5238 (1909 nm)	1908 nm corresponded to O–H stretching first overtone
H	5265 (1899 nm)	1900 nm corresponded to O–H stretching + 2 × C–O stretching of starch
	5230 (1912 nm)	1908 nm corresponded to O–H stretching first overtone
	5180 (1930 nm)	O–H stretching and HOH bending combination (polysaccharides); X-loading peaks at ~5215 cm^−1^ (1918 nm)
	4779 (2093 nm)	2090 nm corresponded to O–H combination
N	4424, 4366 (2260, 2290, 2294 nm)	N–H stretching + C=O stretching of amino acid; important peaks at ~5107 cm^−1^ (1958, 1960 nm N–H asymmetric stretching, amide II)
	5180 (1930 nm)	O–H stretching and HOH bending combination of polysaccharides
	6920 (1445 nm)	N–H primary aromatic amine
O	8470, 8613 (1161, 1180 nm)	1160 nm C=O (carbonyl >C=O) for regression coefficient
	6117 (1635 nm)	C–H vinyl C–H (associated with –CH_2_–CH–)
	5982 (1672 nm)	C–H aromatic C–H (aryl)
	6306 (1586, 1583 nm)	O–H stretching band of alcohol or water (O–H) for X-loading plots
S	5346, 5446 (1836, 1870 nm)	High peaks observed
	5280 (1892, 1894 nm)	O–H bending bonding between water and exposed polyvinyl alcohol OH
	4416, 4424, 5797 (1725, 2260, 2265 nm)	C–H stretching first overtone peaks of X-loading

**Table 5:** GHG emissions and GWP of 1 kg of sugarcane bagasse by combustion, calculated according to IPCC guidelines, and emission of GHGs evaluated by HHV predicted by NIR spectroscopic model.

GHGs	Emission by IPCC (kg [kg Biomass^−1^])	Emission by NIR (kg [kg Biomass^−1^])	GWP by IPCC (kg CO_2_eq)	GWP by NIR (Both by HHV PLSR Model Equal to by GWP PLSR Model) (kg CO_2_eq)
**CO_2_**	0.001687784	0.001714832	0.001687784	0.001714832
**CH_4_**	0.000452085	0.000459330	0.013472133	0.013688034
**N_2_O**	0.000060278	0.000061244	0.016455894	0.016719612
**Total**	0.002200147	0.002235406	0.031615811	0.032122478

**Table 6:** Emission factors (EFs) of sugarcane bagasse-fired boilers in Brazil (^a,b^) compared to the values obtained for sugarcane bagasse samples in this case study by means of the NIR spectroscopic model.

GHG	EF by Reference [104]	EF Used for NIR Prediction ^d^ (kg TJ^−1^)	GWP ^b,c^ (kg CO_2_eq)	CO_2_eq ^b^ (kg CO_2_eq [Ton Bagasse]^−1^)	GWP ^d^ (kg CO_2_eq)	CO_2_eq (kg CO_2_eq [Ton Bagasse]^−1^)	CO_2_eq by NIR (kg CO_2_eq [Ton Bagasse]^−1^)
CH_4_	41.1 ^e^ kg TJ^−1^	30	25	7.74	29.8	13.47	13.69
N_2_O	4.22 ^f^ g mm^−1^ Btu^−1^	4	298	8.98	273	16.46	16.72
Total				16.72		29.93	30.41

^a^ The emission of CO_2_ was not calculated because bagasse is considered to be biomass. ^b^ [104]. ^c^ GWP = global warming potential; based on IPCC (2007) [105]. ^d^ GWP = global warming potential; based on IPCC (2021) [48]. ^e^ Based on MST (2012) [104,106]. ^f^ Based on software: GREET1_2011, emission factor (EF) sheet: sugarcane bagasse/small industrial boiler.

**Table 7:** The higher heating value (HHV) of different species of biomass measured in some research and GHG emissions and IPCC-based GWP calculated by the HHV.

No.	Biomass Sample	HHV (MJ kg^−1^)	CO_2_ Emissions (kg [kg Biomass] ^−1^) × 10^−5^	CH_4_ Emissions (kg [kg Biomass]^−1^) × 10^−5^	N_2_O Emissions (kg [kg Biomass]^−1^) × 10^−5^	IPCC-Based GWP (kg CO_2_eq) × 10^−5^	Source
1	Bagasse	17.5	196.0	52.5	7.0	3671.5	[108] Kumar & Pratt (1996)
2	Rice husk	15.2	170.2	45.6	6.1	3189.0	
3	Rice straw	15.5	173.6	46.5	6.2	3251.9	
4	Wheat straw	16.0	179.2	48.0	6.4	3356.8	
5	Groundnut shell	18.2	203.8	54.6	7.3	3818.4	
6	Coconut shell	20.8	233.0	62.4	8.3	4363.8	
7	Wood	19.6	219.5	58.8	7.8	4114.1	
8	Sawdust	18.9	211.7	56.7	7.6	3965.2	
9	Bark	19.8	221.8	59.4	7.9	4156.0	
10	Beech wood	19.2	215.0	57.6	7.7	4028.2	[109] Demirbaş (1997)
11	Ailanthus wood	19.0	212.8	57.0	7.6	3986.2	
12	Corncob	17.0	190.4	51.0	6.8	3566.6	
13	Corn stover	17.8	199.4	53.4	7.1	3734.4	
14	Sunflower shell	18.0	201.6	54.0	7.2	3776.4	
15	Hazelnut shell	20.2	226.2	60.6	8.1	4238.0	
16	Walnut shell	21.6	241.9	64.8	8.6	4531.7	
17	Olive husk	20.9	234.1	62.7	8.4	4384.8	
18	Eucalyptus leaves	21.0	235.2	63.0	8.4	4405.8	[110] Núñez-Regueira et al. (2001)
19	Eucalyptus branches	18.4	206.1	55.2	7.4	3860.3	
20	Pinus wood	20.2	226.2	60.6	8.1	4238.0	
21	Pinus branches	19.5	218.4	58.5	7.8	4093.1	
22	Shrub (*Erica arborea*)	21.4	239.7	64.2	8.6	4489.7	
23	Poplar wood	19.44	217.7	58.3	7.8	4078.5	[111] Gravalos et al. (2016)
24	Willow wood	19.26	215.7	57.8	7.7	4040.7	
25	Eucalyptus wood	19.83	222.1	59.5	7.9	4160.2	
26	Pine wood	20.12	225.3	60.4	8.0	4221.2	
27	Oak wood	19.97	223.7	59.9	8.0	4189.7	
28	Wheat straw	16.34	183.0	49.0	6.5	3428.1	
29	Barley straw	16.78	187.9	50.3	6.7	3520.4	
30	Corn residues	17.52	196.2	52.6	7.0	3675.7	
31	Sunflower residues	17.89	200.4	53.7	7.2	3753.3	
32	*Quercus robur*	19.73	221.0	59.2	7.9	4139.2	[112] Cordero et al. (2001)
33	*Pinus halepensis*	20.22	226.5	60.7	8.1	4242.2	
34	Eucalyptus sp.	20.08	224.9	60.2	8.0	4212.8	
35	Wheat straw	18.15	203.3	54.4	7.3	3807.8	
36	Olive material	21.06	235.9	63.2	8.4	4418.4	
37	SCG (raw)	21.64	242.4	64.9	8.7	4540.1	[113] Park et al. (2021)
38	SCG hydrochar 180 °C	23.8	266.6	71.4	9.5	4995.2	
39	SCG hydrochar 200 °C	25.1	281.1	75.3	10.0	5265.0	
40	SCG hydrochar 220 °C	26.4	295.7	79.2	10.6	5538.7	
41	SCG hydrochar 240 °C	27.8	311.4	83.4	11.1	5832.4	
42	*Alnus nepalensis*	17.932	200.8	53.8	7.2	3762.1	[94] Shrestha et al. (2023)
43	*Pinus roxburghii*	18.349	205.5	55.0	7.3	3849.6	
44	*Bambusa vulgaris*	17.310	193.9	51.9	6.9	3631.6	
45	*Eucalyptus camaldulensis*	17.105	191.6	51.3	6.8	3588.6	
46	*Bombax ceiba*	17.077	191.3	51.2	6.8	3582.8	
47	*Zea mays* (cob)	17.297	193.7	51.9	6.9	3628.9	
48	*Zea mays* (shell)	16.409	183.8	49.2	6.6	3442.6	
49	*Zea mays* (stover)	16.753	187.6	50.3	6.7	3514.8	
50	*Oryza sativa*	15.417	172.7	46.3	6.2	3234.5	
51	*Saccharum officinarum*	17.029	190.7	51.1	6.8	3572.7	

## Data Availability

No datasets were experimentally generated during the current study.

## Abbreviations

AEDP	Alternative Energy Development Plan
AFOLU	Agriculture, forestry and other land use
ANN	Artificial neural network
AR5	Fifth assessment report
AR6	Sixth assessment report
BTG	Boiler, turbine, and generator technology
C	Carbon
CAGR	Compound annual growth rate
CC	Carbon content
CEM	Continuous emission monitoring systems
C_f_	Combustion efficiency
CH_4_	Methane
CHNOS	Biomass elements, including carbon, hydrogen, nitrogen, oxygen, and sulfur
CNN	Convolutional neural network
COP	Conference of the parties (UNFCCC)
CO_2_	Carbon dioxide
COVM	Covariance matrix
CRDS	Cavity ring-down spectroscopy
QCL	Dual-comb spectroscopy, quantum cascade laser
E	Energy content of fuel burned
EF	Emission factor
EFx	Emission factor of gas x
EPA	Environmental Protection Agency
Ex	Mass of gas x emitted
FTIR	Fourier transform infrared
GC	Gas chromatography
GC-ECD	GC-electron capture detector
GC-FID	GC-flame ionization detector
GC-TCD	GC-thermal conductivity detector
GHG	Greenhouse gas
GWP	Global warming potential
H	Hydrogen
HHV	Higher heating value
IEA	International Energy Agency
iGA	Improved genetic algorithm
IPCC	Intergovernmental Panel on Climate Change
LDC	Least developed country
LHV	Lower heating value
MAE	Mean absolute error
MBE	Mean bias error
N	Nitrogen
NDIR	Non-dispersive infrared
NI-PT-CIMS	Negative-ion proton-transfer chemical ionization mass spectrometry
NZE	Net zero emissions
NIR	Near-infrared
N_2_O	Nitrous oxide
O	Oxygen
OA-ICOS	Off axis integrated cavity output spectroscopy
OF	Fraction of carbon oxidized
OP-FTIR	Open-path Fourier transform infrared spectroscopy
PLSR	Partial least-squares regression
PIT-MS	Proton-transfer-reaction ion-trap mass spectrometry
PTR-MS	Proton-transfer-reaction mass spectrometry
R^2^	Coefficient of determination
R^2^P	Coefficient of determination of prediction
RMSECV	Root mean square error of cross-validation
RMSEP	Root mean square error of prediction
RPD	Ratio of prediction to deviation
S	Sulfur
SDGs	Sustainable Development Goals
SEP	Standard error of prediction
SNV	Standard normal variate
Solar PV	Solar photovoltaics
SVM	Support vector machine
TDLA	Tunable diode laser absorption spectroscopy
TGA/DTA	Thermogravimetric analysis and derivative thermogravimetric analysis
UAV	Unmanned aerial vehicle
UNFCCC	United Nations Framework Convention on Climate Change
